# Convergent evolution of the sensory pits in and within flatworms

**DOI:** 10.1186/s12915-023-01768-y

**Published:** 2023-11-22

**Authors:** Ludwik Gąsiorowski, Isabel Lucia Dittmann, Jeremias N. Brand, Torben Ruhwedel, Wiebke Möbius, Bernhard Egger, Jochen C. Rink

**Affiliations:** 1 Department of Tissue Dynamics and Regeneration, Max Planck Institute for Multidisciplinary Sciences, Am Fassberg 11, 37077 Göttingen, Germany; 2 Institut Für Zoologie, Universität Innsbruck, Technikerstraße 25 6020, Innsbruck, Austria; 3 Electron Microscopy Facility, Department of Neurogenetics, Max Planck Institute for Multidisciplinary Sciences, City Campus, Hermann-Rein-Str. 3, 37075 Göttingen, Germany

**Keywords:** Regeneration, Paratomy, Asexual reproduction, Rhabdomeric photoreceptors, Morphology, Spiralia, Turbellaria

## Abstract

**Background:**

Unlike most free-living platyhelminths, catenulids, the sister group to all remaining flatworms, do not have eyes. Instead, the most prominent sensory structures in their heads are statocysts or sensory pits. The latter, found in the family Stenostomidae, are concave depressions located laterally on the head that represent one of the taxonomically important traits of the family. In the past, the sensory pits of flatworms have been homologized with the cephalic organs of nemerteans, a clade that occupies a sister position to platyhelminths in some recent phylogenies. To test for this homology, we studied morphology and gene expression in the sensory pits of the catenulid *Stenostomum brevipharyngium*.

**Results:**

We used confocal and electron microscopy to investigate the detailed morphology of the sensory pits, as well as their formation during regeneration and asexual reproduction. The most prevalent cell type within the organ is epidermally-derived neuron-like cells that have cell bodies embedded deeply in the brain lobes and long neurite-like processes extending to the bottom of the pit. Those elongated processes are adorned with extensive microvillar projections that fill up the cavity of the pit, but cilia are not associated with the sensory pit. We also studied the expression patterns of some of the transcription factors expressed in the nemertean cephalic organs during the development of the pits*.* Only a single gene, *pax4/6*, is expressed in both the cerebral organs of nemerteans and sensory pits of *S. brevipharyngium*, challenging the idea of their deep homology.

**Conclusions:**

Since there is no morphological or molecular correspondence between the sensory pits of *Stenostomum* and the cerebral organs of nemerteans, we reject their homology. Interestingly, the major cell type contributing to the sensory pits of stenostomids shows ultrastructural similarities to the rhabdomeric photoreceptors of other flatworms and expresses ortholog of the gene *pax4/6*, the pan-bilaterian master regulator of eye development. We suggest that the sensory pits of stenostomids might have evolved from the ancestral rhabdomeric photoreceptors that lost their photosensitivity and evolved secondary function. The mapping of head sensory structures on plathelminth phylogeny indicates that sensory pit-like organs evolved many times independently in flatworms.

**Supplementary Information:**

The online version contains supplementary material available at 10.1186/s12915-023-01768-y.

## Background

Free-living flatworms (also known as “Turbellaria”) occur in diverse environments (marine, freshwater, and terrestrial), which they can perceive with various types of sensory organs. While simple rhabdomeric eyes are the most conspicuous sensory structures in many of the turbellarians, other types of specialized receptive organs can also be found across the phylogeny of platyhelminths. For instance, Catenulida, a relatively small clade of free-living flatworms that occupy a sister position to all the remaining platyhelminths [1–3], do not have pigmented eyes (with a possible exception of the enigmatic *Tyrrheniella sigillata*, which has remained unobserved since its discovery in the 1950s [4]). Instead, members of the catenulid families Retronectidae, Paracatenulidae, and Catenulidae are equipped with a brain statocyst [e.g., [5, 6], while representatives of the family Stenostomidae possess large sensory organs, in the literature referred to as ciliated pits [e.g., [7–12]. Those pits are paired invaginations on the lateral sides of the head, which have an evident neural connection with the brain and which can change their shape with the help of the associated musculature [9, 10, 12]. Although the size, position, and shape of the pits represent one of the most important characteristics used in the taxonomy of Stenostomidae [7, 8, 11], their function remains mysterious. Somehow similar organs (also referred to as ciliated pits or ciliated grooves) have been reported in several other groups of flatworms, e.g., microstomids [13, 14], prorhynchids [15–19], prolecithophorans [20], triclads [21–23], and bothrioplanids [24], yet, their reciprocal homology has not been tested thus far.

Despite the recent progress in resolving animal phylogeny, the sister group of Platyhelminthes remains disputed. Several transcriptome- and genome-based phylogenies suggest gastrotrichs as the sister group of flatworms (forming together a clade called Rouphozoa [25–27]); however, no clear morphological apomorphies are uniting them. On the other hand, the sister position of flatworms and nemerteans (united in the hypothetical clade Parenchymia), which has support in some of the recent molecular phylogenies [28], has been also traditionally supported by morphological characters [e.g., [29–34]. Homology of the ciliated pits of turbellarians (including catenulids) and the cerebral organs (possibly neuroglandular or chemoreceptive structures present in all major clades of nemerteans) was used in the past as one of such morphological traits in the support of the close relation of nemerteans and platyhelminths [13, 35].

While cerebral organs of different groups of Nemertea are well described on the level of morphology and development [e.g., [35–42], and the gene expression in the developing cerebral organs of a nemertean was recently investigated [42], the ciliated pits of turbellarians remain poorly studied. For example, there is no published data on the gene expression in the sensory pits of any of the flatworm species, and for some of the groups, even the fine morphology of the pits remains undescribed. Therefore, to test for the possible homology of platyhelminth sensory pits and cerebral organs of Nemertea, we carried out a detailed analysis of the sensory pits of *Stenostomum brevipharyngium*, a representative of the catenulid family Stenostomidae. Like many other catenulid species [43–46], *S. brevipharyngium* is capable of full head regeneration and asexual reproduction by means of paratomy (which represents its primary reproductive strategy). Hence, in addition to the description of the details of the adult organs, we study their formation during asexual development and head regeneration and compare both processes on the morphological level. We also provide the first gene expression data in catenulids, comparing the expression patterns of transcription factors that have orthologs expressed in the developing nemertean cerebral organs to test for the possible conservation of the molecular patterning of both structures. Finally, we analyze these data in a phylogenetic framework to discuss the evolution of the sensory pits in flatworms and their potential homology to the cerebral organs of nemerteans.

## Results

### General morphology of the sensory pits

**Fig. 1:**
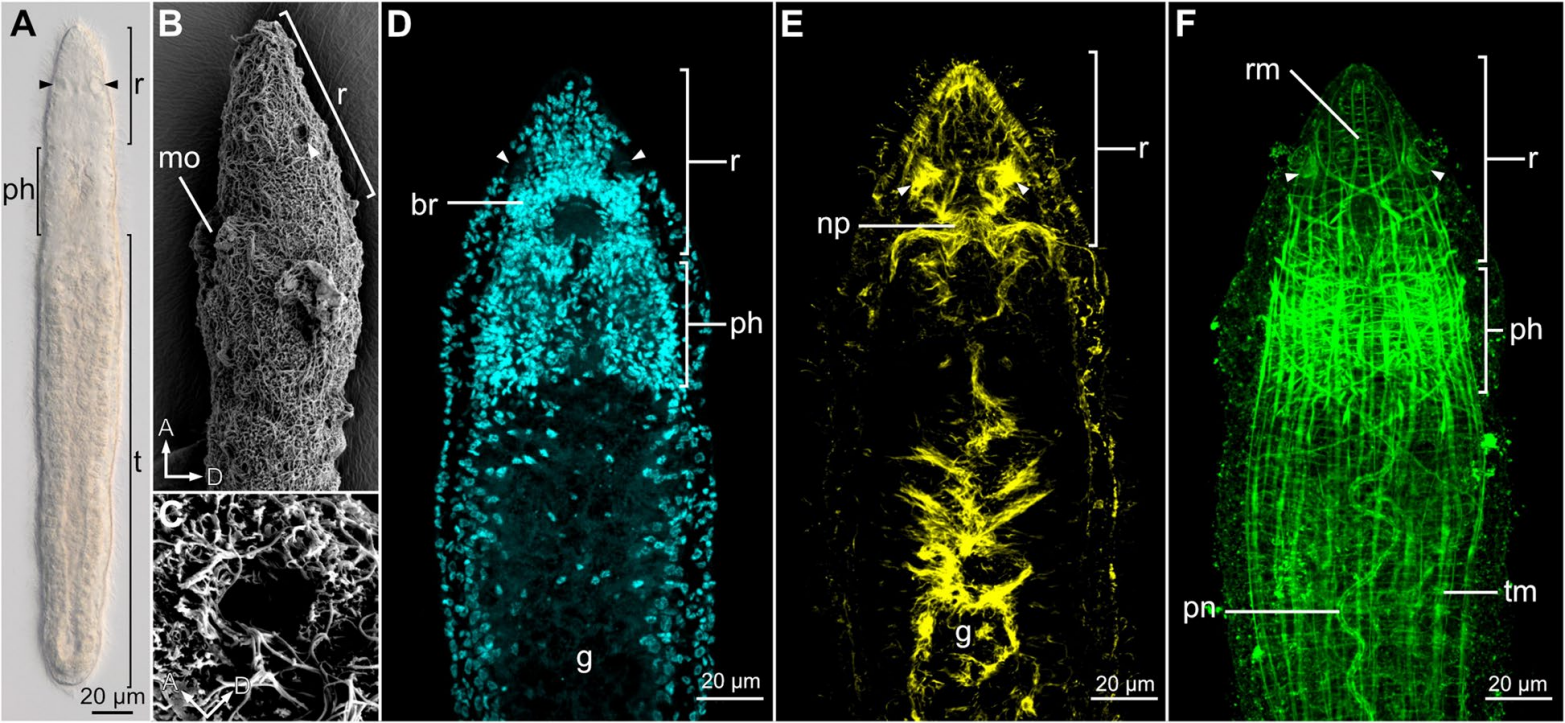
General morphology and localization of sensory pits (arrowheads) in *Stenostomum brevipharyngium*. **A** Light microscopy image of a living worm, showing division into rostrum, pharynx, and trunk. **B** Head region of the worm in the lateral view under scanning electron microscope. **C** Magnified view of the pit opening. Horizontal optical sections through the anterior region of the worm at the level of ciliated pits stained with DAPI for cell nuclei (**D**), antibodies against tyrosinated tubulin (**E**), and phalloidin for F-actin (**F**). Abbreviations: *br* brain, *g* gut, *mo* mouth opening, *np* brain neuropile, *ph* pharynx, *pn* protonephridium, *r* rostrum, *rm* rostral musculature, *t* trunk, *tm* trunk musculature

The body of *Stenostomum brevipharyngium* is divided into the rostrum, pharynx, and trunk (Fig. 1A), and the sensory pits are positioned in the middle of the rostral area. When examined via light microscopy, they appear as two conspicuous globular cavities occupying the midsection of the head (arrowheads, Fig. 1A). Scanning electron microscopy (SEM) clearly reveals the openings of the cavities to the external environment, which are devoid of the otherwise ubiquitous surface cilia (arrowhead, Fig. 1B, C). In confocal laser microscopy (CLM) the sensory pits can be visualized with DAPI staining of cell nuclei (as empty cavities on the lateral sides of the brain, arrowheads, Fig. 1D), with antibody staining against tyrosinated tubulin (arrowheads, Fig. 1E) that reveals ciliated and nervous structures and with phalloidin staining of actin filaments (arrowheads, Fig. 1F).

**Fig. 2:**
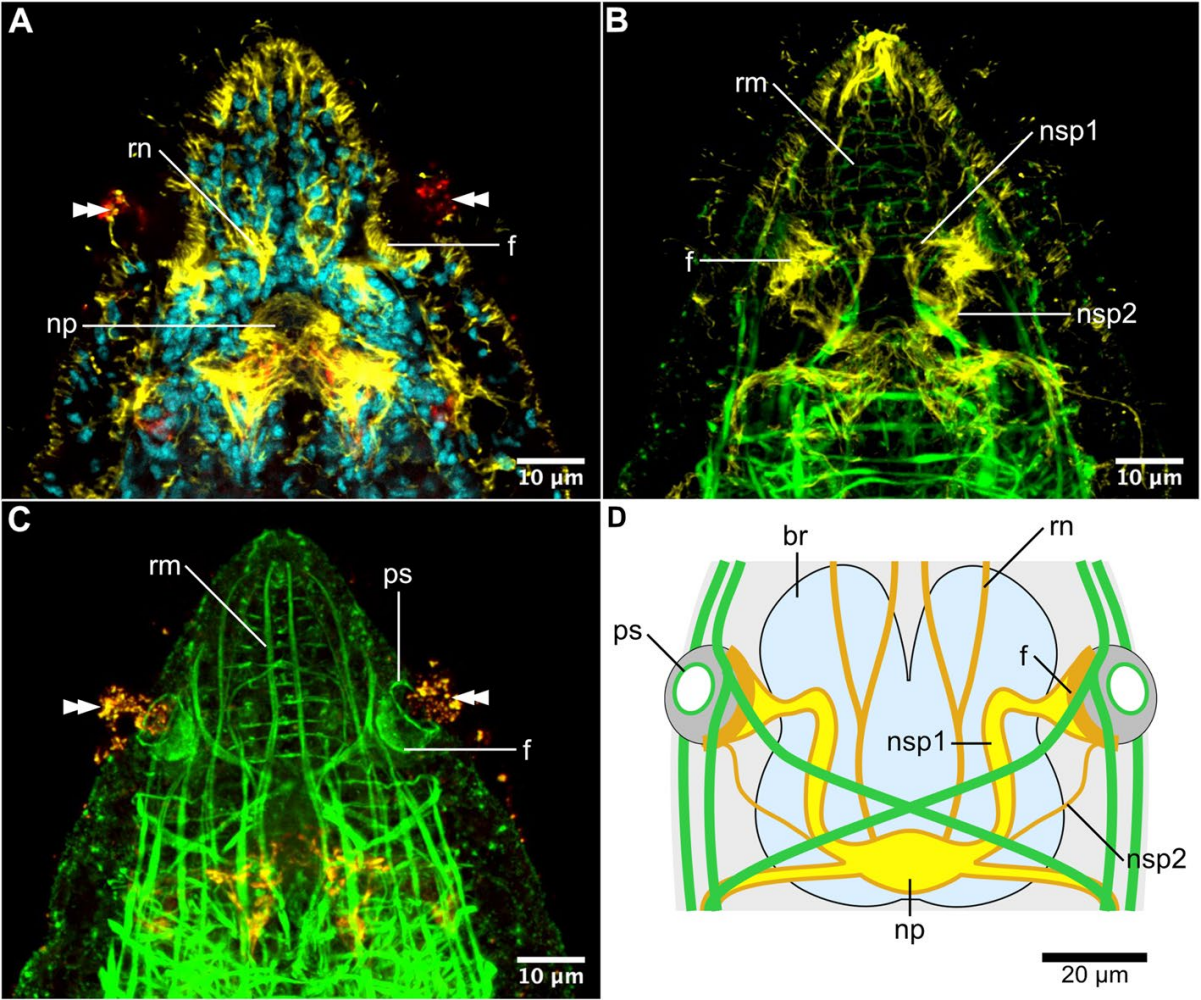
Details of the fully formed sensory pits. **A** Optical horizontal section through the broadest area of the pits showing a fundus, pit cavity, and mucus-like discharge (double arrowheads). **B** Maximum intensity projection showing the nerves of the sensory pit and rostral musculature. **C** Maximum intensity projection showing sphincter and fundus of the sensory pit and mucus-like discharge (double arrowheads). **D** The schematic drawing of the sensory pits in the context of the internal anatomy of the head. Cell nuclei stained with DAPI in *cyan*, a signal from antibodies against tyrosinated tubulin in *yellow*, F-actin stained with phalloidin in *green*, a signal from antibodies against serotonin in *red*. All panels show dorsoventral sections, anterior to the top. Abbreviations: *br* brain, *f* fundus, *np* brain neuropile, *nsp* nerve of the sensory pit, *ps* sphincter of the sensory pit, *rm* rostral musculature, *rn* rostral nerves

Each of the pits is composed of an internal cavity, which laterally opens through the pit opening to the external environment, and the fundus — a bottom part of the pit (Fig. 2). The opening is equipped with a delicate sphincter muscle (*ps*, Fig. 2C and D), that likely controls its width. Although the only muscle fibers that are directly associated with the pits are sphincters, there are numerous additional muscles in the areas adjacent to the organs, such as longitudinal, helical, and circular muscles of the rostrum (Fig. 2B–D). It is likely that those muscles are responsible for the frequent changes in the shape and apparent position of the sensory pits that can be observed in living worms. The fundus can be stained with phalloidin and antibodies against tyrosinated tubulin (*f*, Fig. 2A–C) and does not contain any cell nuclei. Close examination of the tyrosinated tubulin staining reveals continuity between the dense tyrosinated tubulin immunoreactive (tyrTub-IR) projections forming the fundus and neurites that connect the sensory pit with a brain neuropile. The neurites form two bundles, here named nerves of sensory pit 1 and 2 (*nsp1* and *nsp2*, Fig. 2B and D). The nerves are tyrTub-IR and although they follow slightly different paths in the rostrum, they both connect the fundus with the same lateral region of the brain neuropile, just anterior to the root of the main ventrolateral nerve cord (Fig. 2B and D). In several of the fixed specimens, we noticed an apparent secretory discharge from the opening of each of the sensory pits, which was stained with phalloidin and antibodies against serotonin (double arrowheads, Fig. 2A and C). This discharge likely corresponds to the mucus-like substance that has been reported to fill the internal cavities of the pits in *Stenostomum leucops* [9, 10, 12].

### Ultrastructure of the pit

**Fig. 3:**
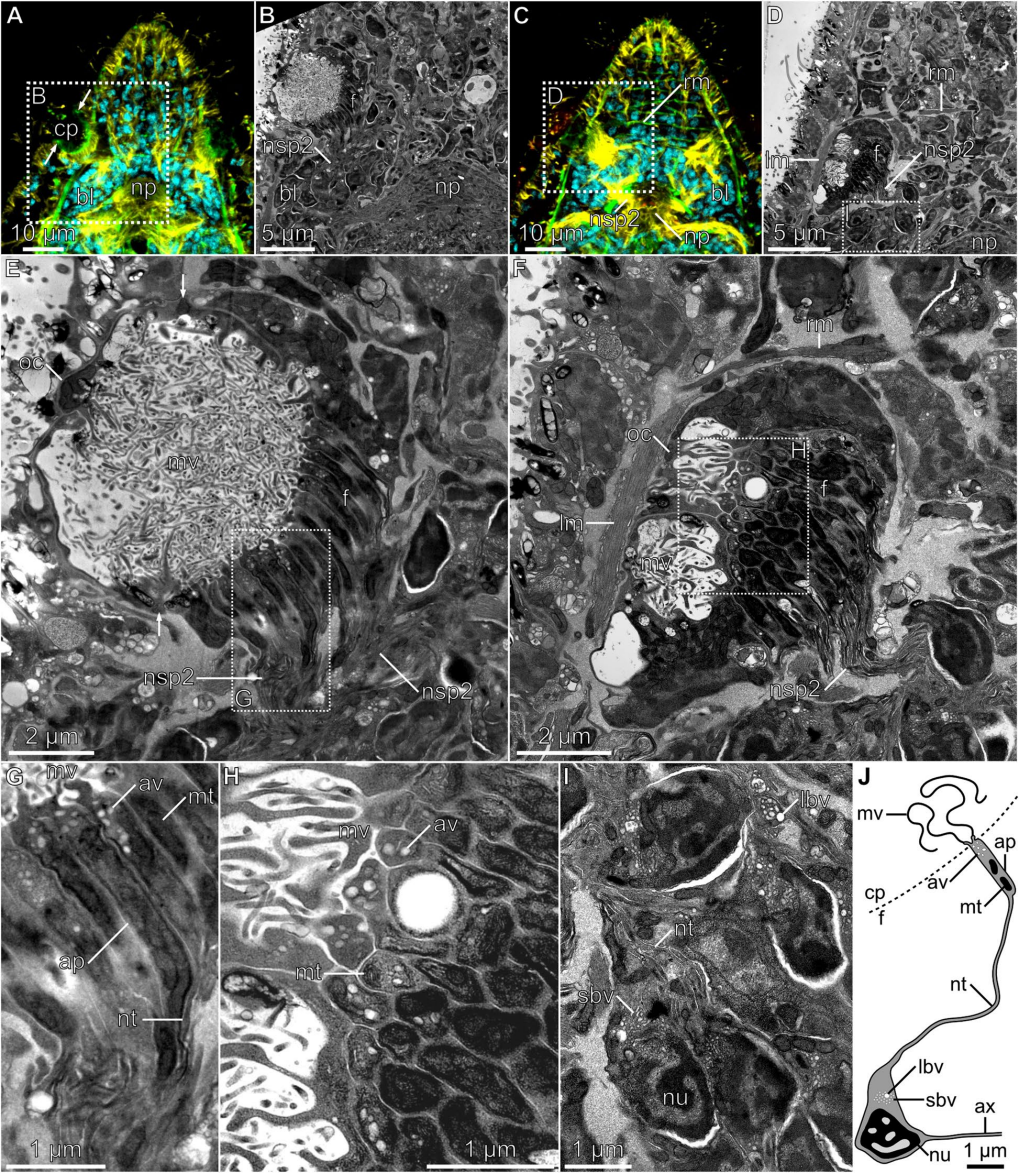
Ultrastructure of the sensory pit. **A** The optical horizontal section showing the approximate area in panel B. **B** The ultrathin horizontal section through the widest part of the sensory pit and associated structures. **C** The optical horizontal section showing the approximate area in panel D. **D** The ultrathin horizontal section through the fundus of the pit and associated structures. **E** Details of the pit as visible in panel B. **F** Details of the fundus as visible in panel D. Longitudinal (**G**) and cross (**H**) section through the cellular processes forming fundus of the pit. **I** Cell bodies of the pit-forming cells as visible in panel D. **J** Schematic reconstruction of the fundus-forming cell. Cell nuclei stained with DAPI in *cyan*, a signal from antibodies against tyrosinated tubulin in *yellow*, and F-actin stained with phalloidin in *green.* The lettered boxes in panels A, C, D, E, and F indicate regions magnified in the corresponding panels. The arrows in panel E indicate muscle fibers of the sphincter. Abbreviations: *ap* apical portion of the fundus cell, *av* apical vesicle, *ax* axon, *bl* brain lobe, *cp* cavity of the pit, *f* fundus, *lbv* large basal vesicle, *lm* longitudinal muscle, *mt* mitochondrion, *mv* microvilli, *np* brain neuropile, *nsp* nerve of the sensory pit, *nt* neurite-like process, *nu* nucleus of the fundus cell, oc outer cell, *rm* rostral musculature, *sbv* small basal vesicle

We next examined the ultrastructure of the pit with transmission electron microscopy (TEM). Specifically, we examined sections at the approximate level of the maximal width of the pit (Sect. 1; Fig. 3A and B) and at its ventral extremity (Sect. 2; Fig. 3C and D).

Section 1 shows that the internal cavity is completely filled with long, convoluted threads, which can be identified as extensive microvilli originating from the surrounding cells (*mv*, Fig. 3E–H). Although the opening of the pit is not present in Sect. 1, the sphincter muscle is visible in the cross-section (arrows, Fig. 3E), as expected from the corresponding optical section obtained with CLM (arrows, Fig. 3A).

The fundus of the pit consists of elongated cell processes which were cut longitudinally in Sect. 1 (*f*, Fig. 3E, G) and transversely or obliquely in Sect. 2 (*f*, Fig. 3F and H). Those densely arranged processes apically give rise to the aforementioned extensive microvilli (Fig. 3G and H). Additionally, their apical regions are packed with numerous small apical electron-translucent vesicles (*av*, Fig. 3G, H, and J). Slightly below the apical vesicular region, conspicuous mitochondria are filling the process (*mt*, Fig. 3G, H, and J). Approximately 1.5 μm below the fundus surface, the cellular processes are becoming considerably thinner and start to extend as neurite-like fibers that form the nerves of the sensory pit (*nt* and *nsp2*, Fig. 3B, D–G). These neurites connect on the other side of the nerve to the bodies of neuron-like cells residing in the lateral lobes of the brain. Although we cannot directly trace the connection between particular brain perikarya and cellular processes at the fundus of the pit, we suggest that the cell bodies (with nuclei) of the fundus-forming cells are located in the lateral parts of the brain lobes. Such an arrangement would explain the lack of cell nuclei in the fundus of the pit that we observed with both CLM and TEM. Besides the nucleus, those cell bodies also contain large and small electron-translucent vesicles (*lbv* and *sbv*, Fig. 3i and J), grouped together close to the base of the neurite-like projection.

Based on these results, we reconstructed the fundus-forming cells as resembling neurons (Fig. 3J). The cell body, containing the nucleus and basal vesicles, is located in the lateral lobes of the brain and sends out neurite-like projection, which forms the nerves of the sensory pit. Apically, the projection swells, forming a structural unit of the fundus, that harbors mitochondria and apical vesicles, and which also gives rise to the modified microvilli that fill the cavity of the pit. The results of antibody stainings suggest that both neurite-like projections (*nt*) and apical portions of these cells (*ap*) are reinforced with tyrosinated tubulin fibers. We could not detect axons emanating from those cells in our sections. However, as antibody staining revealed continuous neural connections between the fundus of the sensory pit and the neuropile, it is likely that the cells also project axons to the neuropile.

The outer walls of the pit (lateral to the edges of the fundus) are formed by the outer cells that also have extensive microvilli (*oc*, Fig. 3E, F), but otherwise do not resemble the fundus cells. A nucleus is the only internal organelle that can be unequivocally identified within those extremely thin cells. Although it is difficult to ascertain, the fiber of the sphincter muscle seems to be also associated with the outer cell (arrows, Fig. 3E).

Altogether our data suggest that the sensory pit of *S. brevipharyngium* is mostly composed of only two cell types — the outer cells, and the fundus-forming cells (that are partially positioned in the brain and also form the nerves of the sensory pit). Importantly, neither of those cell types contains elements of the ciliary apparatus and there are no cilia within the inner cavity of the pit, as evident from antibody staining and TEM sections.

### Formation of the pits during asexual development

**Fig. 4:**
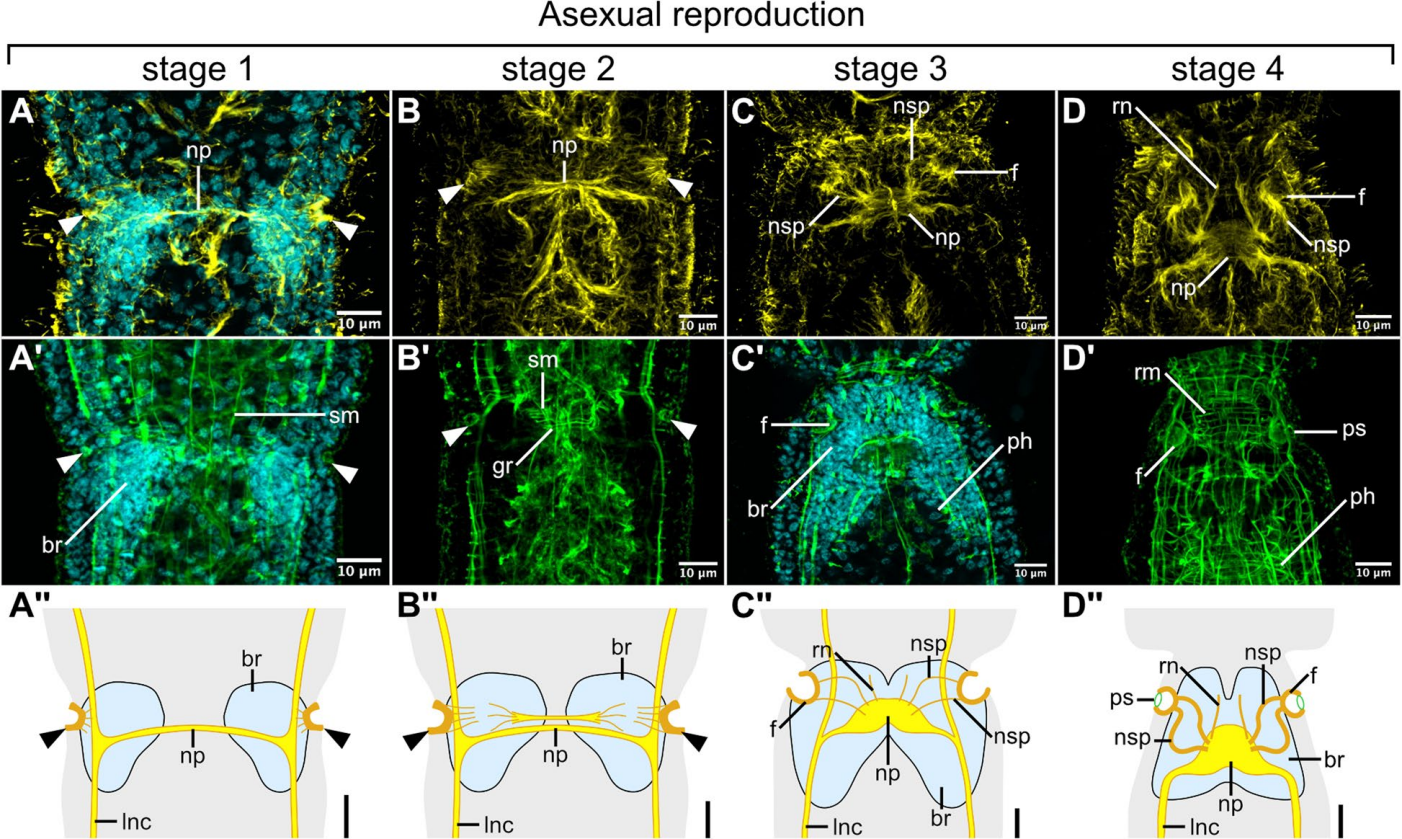
Formation of the pits during asexual development, as visualized with antibodies against tyrosinated tubulin (*yellow*, **A**–**D**), phalloidin staining for F-actin (*green*, **A′**–**D′**), and Hoechst staining for cell nuclei (*cyan*). Schematic drawings of the pit formation (**A″**–**D″**), scale bars 10 μm. Abbreviations: *br* brain, *f* fundus, *gr* reorganizing gut tissue, *lnc* longitudinal nerve cord, *np* brain neuropile, *nsp* nerve of the sensory pit, *ph* pharynx, *ps* sphincter of the sensory pit, *rm* rostral musculature, *rn* rostral nerves, *sm* splanchnic musculature

The asexually reproducing individuals of *S. brvipharyngium* can be distinguished in cultures by longer trunks and an inconspicuous transverse furrow that bisects the trunk at the site of the future fission plane. We used Hoechst, phalloidin, and tyrosinated-tubulin staining to visualize the internal structures of the developing zooid and to stage the temporal sequence of the fission process. At the earliest stage 1 (Fig. 4A), two cell accumulations on each side of the gut and a thin tyrTub-IR commissure connecting the lateral nerve cords constitute the earliest anlage of the future head. At stage 2 (Fig. 4B), the future neuropile thickens via the addition of further neurites, the gut becomes considerably constricted, and the remodeling of splanchnic musculature in the future rostral region becomes evident. In the following stage 3 (Fig. 4C), the brain gains a bilobed shape with the neuropile already resembling its final form. The rudiment of the newly formed zooid already adopts the shape reminiscent of the final head, while the rostrum and pharynx are still undergoing development. At stage 4 (Fig. 4D) the rostral and pharyngeal musculature is established and the general appearance of the zooid rostrum resembles that of a fully formed worm, although it is proportionally smaller. Stage 4 is directly followed by fission between the maternal and new zooids.

The earliest rudiments of the sensory pits can be already observed at stage 1 (arrowheads Fig. 4A), when some of the epidermal cells at the level of the division furrow form small depressions that show strong tyrosinated tubulin immunoreactivity and weaker staining with phalloidin. The short tyrTub-IR projections fan out from these depressions towards developing brain rudiments. At stage 2, the depressions become slightly deeper, the tyrTub-IR projections extend further towards the forming brain neuropile and the phalloidin staining at the prospective fundus area becomes stronger (Fig. 4B). At stage 3, the pits already acquire the form of large empty cavities with a clearly visible fundus, while some of the tyrTub-IR projections reach the neuropile and thus establish the nerves of the sensory pits (Fig. 4C). Already at this stage, there are no cell nuclei associated with the fundus of the pit. Finally, at stage 4, the sphincters of the pit opening become evident in phalloidin staining (Fig. 4D) and the pits take their final form with a contractile opening, inner cavity, fundus, and nerves.

### Formation of the pits during head regeneration

**Fig. 5:**
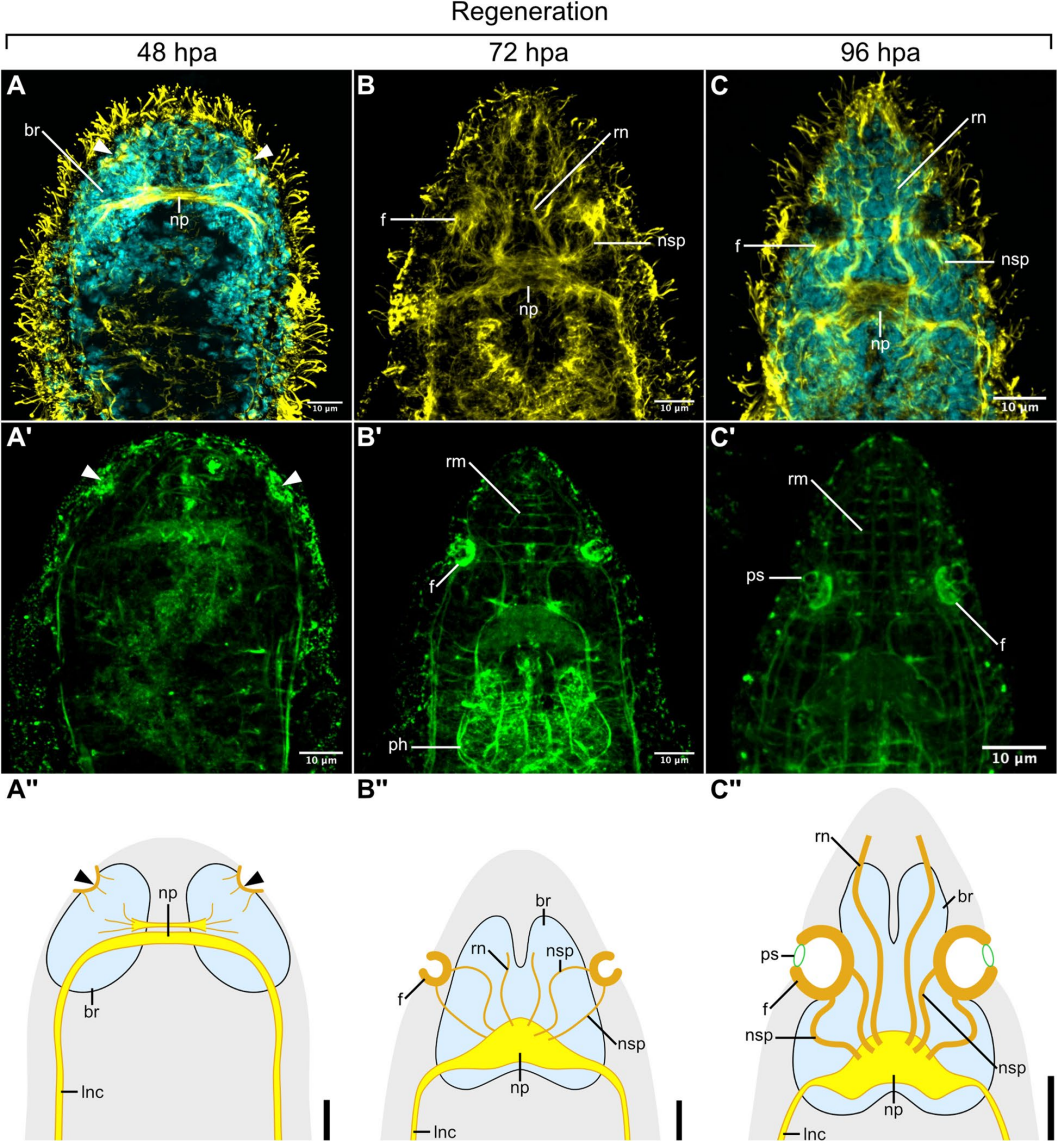
Formation of the pits during head regeneration, as visualized with antibodies against tyrosinated tubulin (*yellow*, **A**–**C**), phalloidin staining for F-actin (*green*, **A′**–**C′**), and Hoechst staining for cell nuclei (*cyan*). Schematic drawings of the pit formation during regeneration (**A″**–**C″**), scale bars 10 μm. Abbreviations: *br* brain, *f* fundus, *lnc* longitudinal nerve cord, *np* brain neuropile, *nsp* nerve of the sensory pit, *ph* pharynx, *ps* sphincter of the sensory pit, *rm* rostral musculature, *rn* rostral nerves

Head regeneration in *S. brevipharyngium* takes about 4 days to complete. The first indication of the formation of sensory pits can be observed at 48 h post-amputation (hpa). At this regeneration stage, the brain rudiment with two lobes and the main commissure of the neuropile is already formed (Fig. 5A). The rudiments of the pits are visible as small invaginations on the surface of the regenerating head that could be stained with phalloidin and antibodies against tyrosinated tubulin (arrowheads, Fig. 5A). Some fine tyrTub-IR projections extend from these invaginations, penetrating the brain rudiment. At 72hpa, the sensory pits are already divided into an internal cavity and fundus that can be visualized with phalloidin and tyrosinated tubulin staining (Fig. 5B). At this stage of head regeneration, the brain neuropile has already reached its final shape and the thick tyrTub-IR nerves of sensory pits can be detected, connecting the neuropile with the pits (nsp, Fig. 5B). At 96hpa, when most of the head structures are already fully regenerated, the sphincter of the sensory pit opening can be visualized by phalloidin staining (Fig. 5C), thus constituting the last regenerating structure of the organ.

### Gene search

To study gene expression in the sensory pits of *S. brevipharyngium* we first needed to generate a reference transcriptome for this species. We pooled together thousands of worms at different stages of asexual development and regeneration, generated the cDNA library, and sequenced it using the Illumina next-generation sequencing method (see the “Methods” section for details). The raw reads were de novo assembled using an established pipeline [47] into the reference transcriptome of *S. brevipharyngium* (see method section for details), which contained 35,979 unique transcripts. We searched this transcriptome using sequences of the six transcription factors (TFs): *otx*, *pax4/6*, *dach*, *tll*, *emx*, and *svp*, which in the nemertean *Lineus ruber* have been shown to be expressed in the developing cerebral organs [42]. The BLAST search identified 10 unique sequences that were confirmed as putative orthologs of those six candidate genes by reciprocal BLASTP search against the NCBI database of protein sequences (Additional file 1: Table S1). The identity of each of those TFs has been further confirmed by the phylogenetic analysis of the protein sequence (Additional file 2: Figs. S1–S5). Four out of those six TFs (*otx*, *pax4/6*, *tll*, and *emx*) have been duplicated in *S. brevipharyngium,* resulting in a total of ten gene targets for expression analysis.

To test whether the sensory pits of *Stenostomum* might have a photoreceptive function, we also searched the *S. brevipharyngium* transcriptome and published transcriptomes of *S. leucops* and *Paracatenula* sp. for opsin gene homologs. Despite the use of different opsin gene sequence queries (see the “Methods” section for details), we were unable to detect opsin homologs in any of the catenulid transcriptomes. The closest BLAST hits always corresponded to other G-protein coupled receptors (e.g., neuropeptide Y receptor, 5-HT receptor, octopamine receptor, tachykinin receptors, dopamine receptor D_2_, neuropeptide F receptor, and muscarinic acetylcholine receptor (Additional file 1: Table S1)), indicating that opsin may have been lost in the examined catenulids.

### Gene expression

**Fig. 6:**
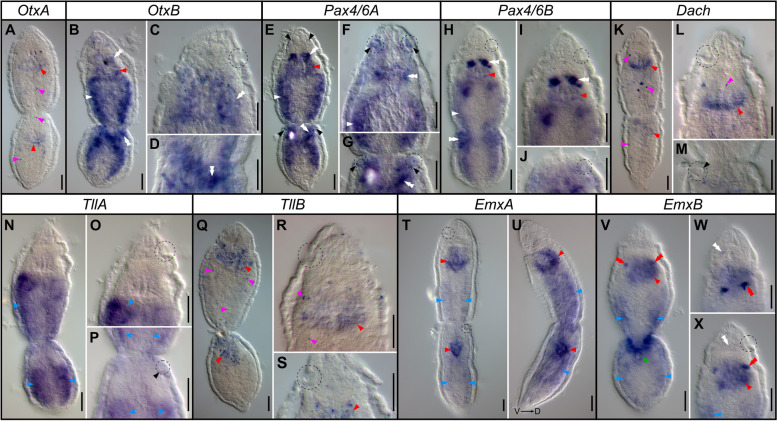
Colorimetric RNA in situ hybridization of the studied transcription factors in the worms in the asexual phase. For each panel, the name of the hybridized gene is indicated in the box above. Sensory pits are marked with a dotted circle. The expression was detected in the following morphological structures: pharynx (red arrowheads), brain (double white arrowheads), longitudinal nerve cords (white arrows), gut tissue (blue arrowheads), distinct pharyngeal domains (double red arrowheads), around the mouth opening (green arrowhead), single cells spread throughout worm body (magenta arrowheads) and in the sensory pits (black arrowheads). Worms are mounted dorsoventrally in all panels with the exception of the laterally mounted animal in panel U. Scale bars 20 μm

To gain insights into the molecular specification of the sensory pits, we examined the expression of the ten identified orthologs of the candidate transcription factors (TFs), *otx*, *pax4/6*, *dach*, *tll*, *emx*, and *svp.* For all of the genes, with the exception of *svp*, we performed colorimetric in situ hybridization (CISH) on the worms in an asexual phase. The genes were expressed in multiple cell types/tissues: in the pharynx (genes: *otxA*, *otxB*, *pax4/6A*, *pax4/6B*, *tllB*, *emxA*, and *emxB*; red arrowheads, Fig. 6B, E, H, I, K, L, Q–X), in the brain (genes: *otxB*, *pax4/6A*, *pax4/6B, and emxB;* double white arrowheads, Fig. 6B–I, W, X), in the longitudinal nerve cords (genes: *otxB*, *pax4/6A* and *pax4/6B*; white arrows, Fig. 6B, E–H), in the gut tissue (genes: *tllA*, *emxA*, and *emxB*; blue arrowheads, Fig. 6N–P, T–V, X), in the additional distinct pharyngeal domains (gene: *emxB*, double red arrowheads, Fig. 6V–X), around the mouth opening (gene: *emxB*, green arrowhead, Fig. 6V) and in individual cells spread throughout the worm body without any clear pattern (genes: *otxA*, *dach*, and *tllB*; magenta arrowheads, Fig. 6A, K, L, Q, R). However, from all of the tested TFs, only *pax4/6A* showed expression in the sensory pits, both in the fully formed and in the developing organs (black arrowheads, Fig. 6E–G).

**Fig. 7:**
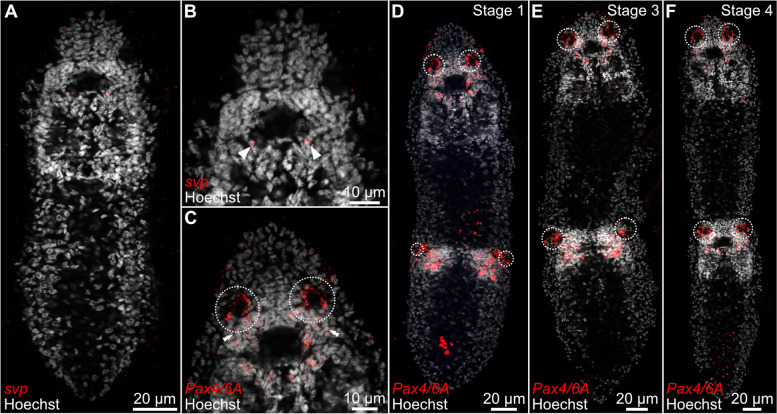
Expression of the genes *svp* and *Pax4/6* as visualized with the RNA in situ hybridization chain reaction. For each panel, the name of the hybridized gene is indicated in the bottom left corner. Gene expression in the sensory pits is marked with a dotted circle

Next, we studied the expression of the genes *svp* and *pax4/6A* with RNA hybridization in situ chain reaction (HCR), which allows visualization of the gene expression at the cellular level. The gene *svp* is expressed only in two neurons residing close to the posterior part of the neuropile (Fig. 7A and B). In the head region, the signal from *pax4/6A* could be detected in the fundus of the sensory pits, in the brain, including the areas in which the cell bodies of the fundus-forming cells are likely located, and in the cells that form sensory pits at the different stages of their development in the asexual zooids (Fig. 7D–F).

### Ancestral state reconstruction

Finally, to test whether the sensory pits of *Stenostomum* are homologous to the similarly positioned ciliated pits of other flatworms we performed ancestral state reconstruction. First, we inferred the phylogeny of flatworms using four molecular markers (*18S*, *28S*, *COI*, and *ITS-5.8S*) that are available for a wide range of platyhelminths including multiple catenulid species and other groups where those organs have been reported (Additional file 1: Table S2). Our species selection aimed at 1. covering evenly all major clades of flatworms, 2. including representatives of the groups for which sensory organs were reported, and 3. choosing species for which a maximum number of molecular markers was available.

The obtained maximum likelihood tree (Additional file 2: Fig. S6) has high support values for major clades of flatworms and shows a similar topology to the already published transcriptome-based phylogenies of platyhelminths [1, 2]. For instance, we recovered the sequential branching of Catenulida, Macrostomorpha, and (Polycladida + Prorhynchida) from the remaining flatworms, the monophyly of Neodermata and Adiaphanida (Additional file 2: Fig. S6). We also recovered the internal topologies of important clades congruent with the published phylogenies of those groups — e.g., (Stenostomidae + (Catenulidae + Paracatenulidae)) within catenulids [3, 11, 48], (Haplopharyngidae + (Macrostomidae + Dolichomicrostomida)) within Macrostomorpha [49], and (Maricola + (Geoplanoidea + Planarioidea)) within triclads [50–52]. A few differences from the topologies of the transcriptome-based phylogenies (e.g., position of bothrioplanids, proseriates, and rhabdocoels), are likely the effect of insufficient phylogenetic signal in the limited number of molecular markers. However, they should not have profound effects on overall ancestral state reconstruction, especially at the base of the tree.

**Fig. 8:**
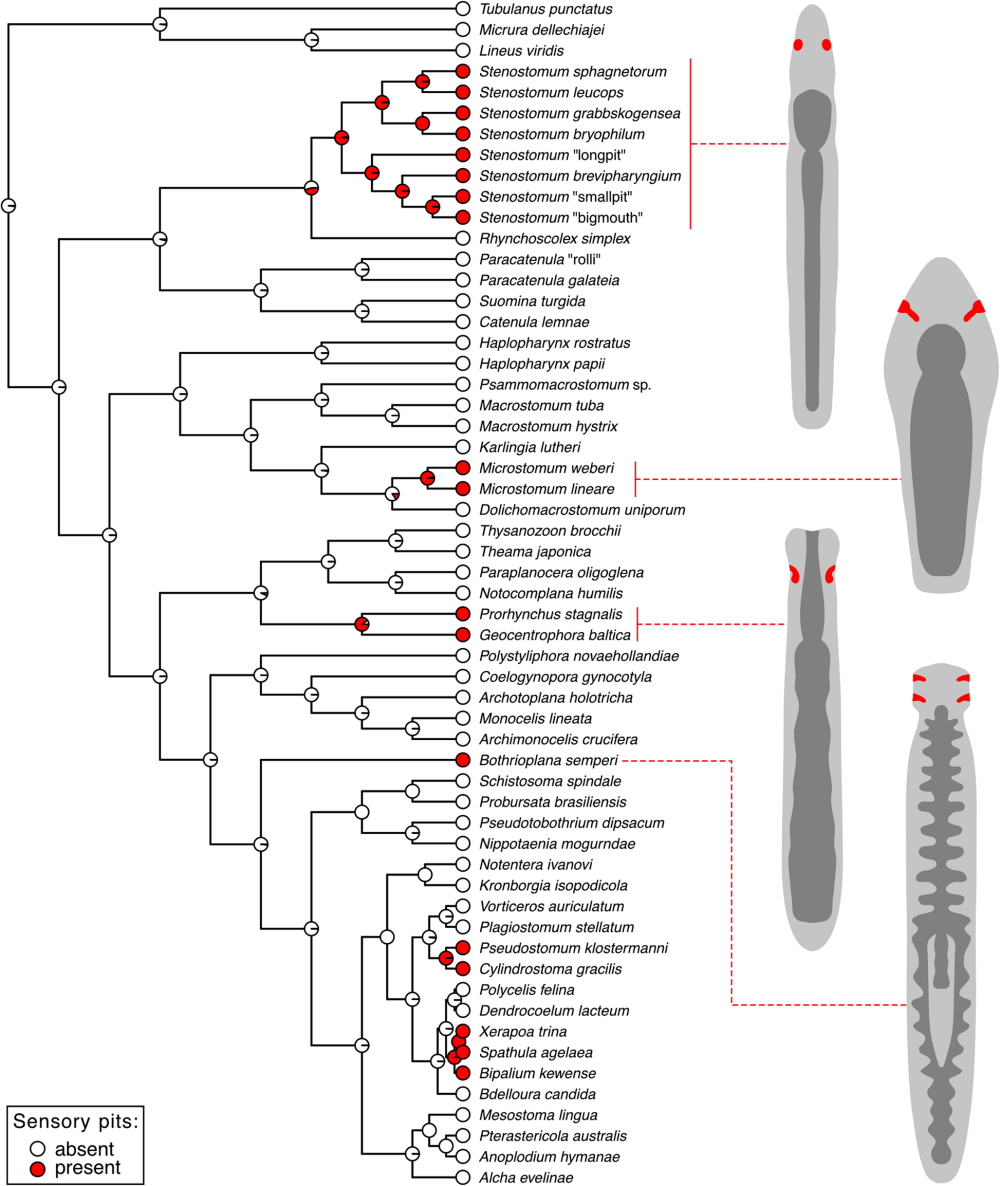
The ancestral state reconstruction of the presence of the sensory pit-like organs in flatworms. The tree topology was inferred based on a maximum-likelihood analysis of concatenated *18S*, *28S*, *ITS-5.8S*, and *COI* datasets (Additional file 2: Fig. S6). The pie charts show the likelihood of the character states at each node. Schematic drawings of the chosen representative flatworms show the position of their sensory pit-like organs (in *red*) in relation to the body outline (*light gray*) and gut (*dark gray*)

Based on the available literature we scored the presence or absence of the sensory/ciliated pits in different species included in our analysis and performed ancestral state reconstruction using stochastic character mapping (see the “Methods” section for details). Our results clearly indicate that sensory/ciliated pits evolved at least six times independently within flatworms — in stenostomids, microstomids, prorhynchids, bothrioplanids, pseudostomids, and geoplanoids (Fig. 8), refuting the hypothesis of their reciprocal homology.

## Discussion

### Sensory pits of *Stenostomum*

In the literature, the head organs of Stenostomidae are referred to as “ciliated pits” [7–12]. However, as we demonstrated here, the organs are entirely devoid of any discernible ciliation in *S. brevipharyngium* and in fact, their most common cellular component in other *Stenostomum* species is also not ciliated [9, 10]. Therefore, we propose to abandon the misleading term “ciliated pits” and instead refer to those structures as “sensory pits”.

Although the sensory pits represent one of the most prominent morphological structures of Stenostomidae that are important in the taxonomy of the family [7, 8, 11], there are relatively few studies on their detailed morphology and development. The formation of the organs during asexual reproduction and their ultrastructure have been investigated so far only in *Stenostomum* cf. *leucops* [9, 10, 12]. We found several similarities between the pits of *S. brevipharyngium* and *S. leucops*, e.g., the absence of cilia in the fundus-forming cells [9, 10], indications of a mucus-like substance filling the internal cavity of the organ [9, 10, 12], the presence of enlarged microvilli on the fundus-forming cells [9] and connection of the organs to the brain by prominent nerves [9, 10]. However, we also noted some differences that could be attributed either to the improved observation techniques or to interspecific differences.

Kepner and Cash [10] described musculature associated with the sensory pit in *S. leucops*. They mention that the mass of the pit (which they refer to as the “ganglion of the ciliated pit,” see below) is separated from the brain by muscle fibers that encapsulate the pit. In *S. brevipharyngium*, we were not able to detect the corresponding musculature, neither with CLM nor with TEM. Instead, we found numerous rostral muscles, structurally independent of the pit, that penetrate the brain mass in the close vicinity of the fundus. It seems likely that the corresponding muscles have been mistakenly interpreted by Kepner and Cash as the muscular capsule of the pit. Additionally, we found that the opening of the pit is supported by the delicate sphincter, which has not been reported in *S. leucops*. The aforementioned “ganglion of the ciliated pit” in *S. leucops* is another structure that we could not confirm in *S. brevipharyngium*. According to Kepner and Cash, this “ganglion” is composed of the neurons, that send their projections to the pit, but otherwise do not differ from the brain neurons and are only separated from the rest of the brain by the musculature of the pit. We also found neuron-like cells sending their projections to the fundus of the sensory pits, however, those neurons were not separated from the rest of the brain by any musculature (as discussed above) and instead, they represent a structurally integral part of the brain. The study of Kepner and Cash was performed using histological sections, which in the case of microscopic animals are less informative than TEM and CLM. Taking into account that with the latter techniques, we were able to find minute structures that were not mentioned in Kepner and Cash (e.g., the sphincter of the sensory pits), it is unlikely that we missed any major structures in our investigation. Therefore, we suspect that both the muscular capsule surrounding the pits and the ganglion of the pit are artifacts, resulting from misinterpretation of the histological sections.

The TEM study by Reuter et al. [9] identified three cell types within the pits of *S. leucops*: the most numerous type I cells have extensive microvilli and deeply positioned nuclei but lack cilia, type II cells have short aberrant cilia, while type III cells have cilia originating from small cylindrical invaginations surrounded by collars of microvilli. In *S. brevipharyngium*, we were able to detect only a single cell type within the fundus of the organ, which shows many ultrastructural similarities to the type I cells described by Reuter et al. Since we were not able to detect any cilia within the cavity of the pit (both with CLM and TEM) we believe that the type I-like cells are the only ones present in the fundus of *S. brevipharyngium*. The other cell type that we could observe in the pit — the outer cells of the pit — has not been reported by Reuter et al. We propose that the difference in the cell type composition between our study and the one by Reuter et al. likely reflects interspecific variation. *S. leucops* is generally larger than *S. brevipharyngium* and also has much more prominent pits that are widely opened to the exterior [8, 12]. Therefore, it is possible that either new sensory cell types were added to the enlarged sensory pits of *S. leucops* or that some of the ancestral cell types were lost in *S. brevipharyngium*, due to the miniaturization of its pits. On the other hand, the outer cells and sphincter of the pit in *S. brevipharyngium* may represent morphological specialization of the constricted pits, present in *S. brevipharyngium* but lacking in *S. leucops*. Nevertheless, in both species, the most prevalent cell type structurally contributing to the organ is very similar — the neuron-like cells with cell bodies located deeply in the brain, a neurite-like process extending towards the fundus, and a distal portion located in the fundus that has numerous mitochondria, dense microtubular skeleton, and extensive apical microvilli. The homology of this cell type between the two *Stenostomum* species seems well supported.

The formation of sensory pits during asexual development has been described in detail for *S. leucops* [10, 12]. In this species, the organs appear to originate from epidermal cells that lose cilia and then migrate toward the brain, which develops from another cellular source (likely the neoblast-like cells located between the gut and the epidermis). Our observation supports a similar pattern in *S. brevipharyngium*: at the earliest stage of development, the ciliated pits are composed of small invaginations of epidermal cells, later the cell nuclei move deeper towards the developing brain lobes and finally integrate with them, leaving behind the neurite-like processes that form nerves of the sensory pits. A similar sequence of morphogenetic events could also be observed during head regeneration, indicating that the formation of sensory pits follows a similar pattern irrespective of whether it is part of the asexual reproduction cycle or regeneration.

### Are sensory pits derived from the photoreceptive organs?

**Fig. 9:**
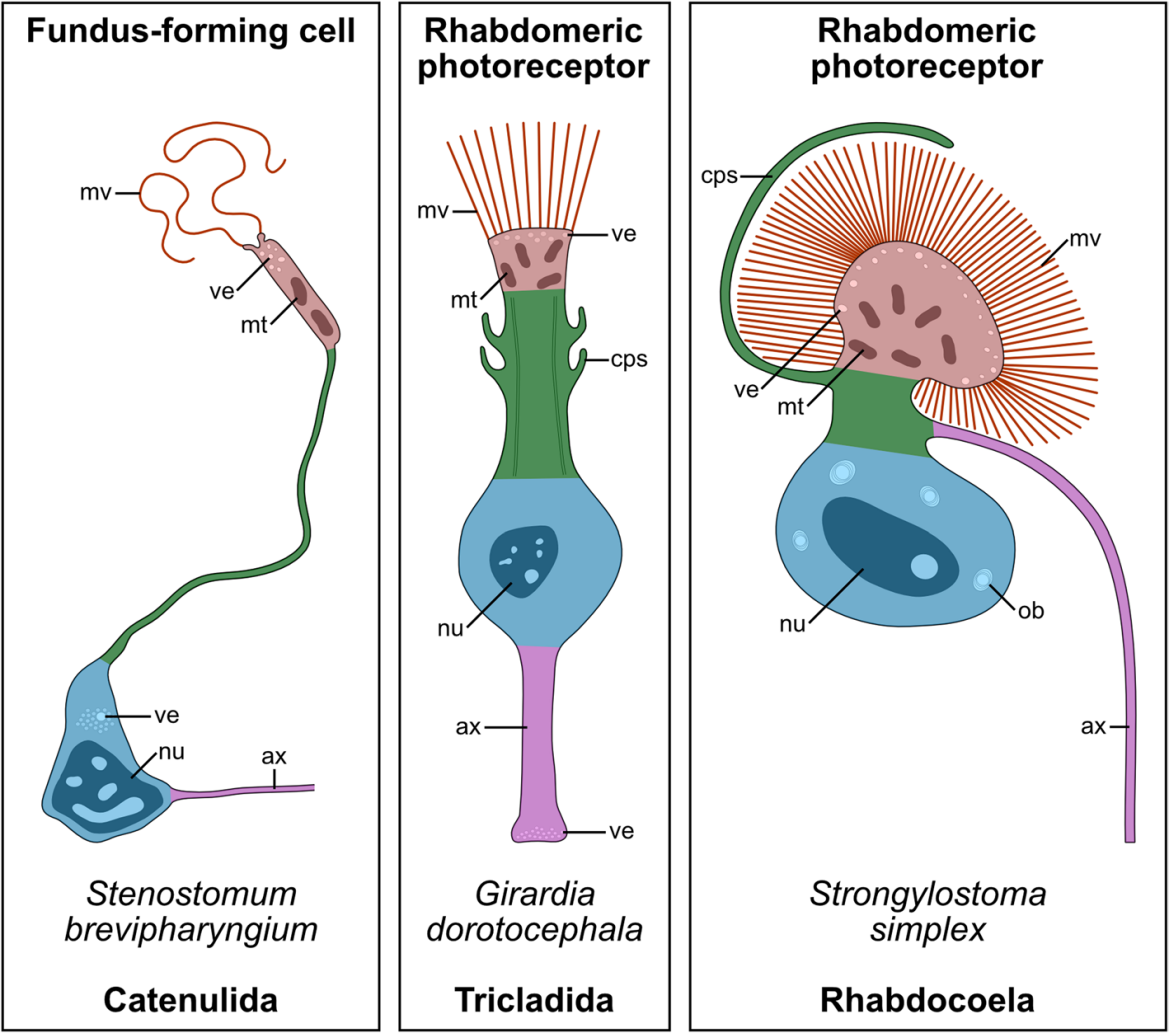
Proposed homology of the fundus-forming cell of *Stenostomum* and rhabdomeric photoreceptors of Rhabditophora. Each of the cells can be divided into four corresponding regions showing positional and ultrastructural similarities: the apical microvillar region (in *red*) is equipped with prominent microvilli and harbors numerous vesicles and mitochondria, the microvillar region is connected by the neck region (in *green*) to the nuclear region (in *blue*), that is positioned outside of the receptive organ, finally, the cell connects to the brain neuropile by axon (in *magenta*). Drawings of rhabdomeric photoreceptors after Carpenter et al. (1974) [55] (for triclads) and Bedini and Lanfranchi 1998 [54] (for rhabdocoels). Abbreviations: *ax* axon, *cps* cytoplasmic processes, *mt* mitochondria, *mv* microvilli, *nu* nucleus, *ob* onion bodies, *ve* vesicles

The exact function of the sensory pits of *Stenostomum* remains unknown, but chemoreceptive or mechanoreceptive roles have been suggested on the basis of their morphology [9, 10]. Here, we additionally would like to point out the striking similarities between the sensory pits of the catenulids and the photoreceptors of other flatworms (united in the clade Rhabditophora). The eyes of most free-living rhabdiotophorans are composed of pigmented cup cells and rhabdomeric photoreceptors [e.g., [53–58]. In general, the photosensory cells of Rhabditophora are neuron-like, composed of somata closely associated with the brain, and elongated neck-like processes that extend toward the eye cups (Fig. 9). These cellular extensions are reinforced with microtubules, distally they harbor numerous mitochondria and vesicles, and at their apical surface, they exhibit numerous extensive microvilli (the so-called rhabdomeres) that fill the empty cavity of the concave pigmented cup and thus enlarge the photoreceptive surface. Therefore, in many aspects, the rhabdomeric photoreceptors of Rhabditophora are structurally similar to the fundus type I cells of Stenostomidae, both being composed of the corresponding regions with similar ultrastructure and relative position (Fig. 9). The resemblance between the sensory pits of *Stenostomum* and the eyes of rhabditophorans goes beyond the cell type composition. Both organs are cup-shaped with the internal cavity filled by the microvilli, have a similar position in the head, and connect directly to the brain with prominent nerves. Moreover, we found that *pax4/6A*, an ortholog of the bilaterian master regulator of eye development [59–61], is also expressed in the fundus-forming cells of *Stenostomum*, further strengthening the hypothesis that this cell type might be derived from a photoreceptor. Although eye formation in triclads is *pax4/6*-independent [62, 63], the loss of the eye-patterning function of this gene is a derived character that might have evolved after the split of Catenulida from the lineage of Rhabditophora.

There are some indirect and anecdotal indications that members of Stenostomidae are capable of photoreception ([64–66], LG personal observations, Dian-Han Kuo personal communication). However, the likely photoreceptive cells, the so-called “light-refracting bodies” of *Stenostomum* are located outside the sensory pits [65, 66] and we were not able to identify any opsins in *S. brevipharyngium* nor in any of the other available catenulid transcriptomes (*Stenostomum leucops* and *Paracatenula* sp.). Similarly, antibodies against opsin of *Dugesia japonica* did not reveal any significant immunoreactivity in *Stenostomum* sp. [67] and the lack of opsins in Catenulida has been reported as part of a phylum-wide systematic transcriptome search for flatworm opsin genes by Rawlinson et al. [57]. Altogether, these observations indicate that stenostomid photoreception is unlikely to be associated with opsins in the rhabdomeric receptors and, if present at all, it is likely associated with yet unknown molecular and cellular mechanisms.

The possibility of the functional shift from photosensitivity to mechanoreception in animals has been put forward in the past [68],﻿ and recently it has been demonstrated for the rhabdomeric non-cephalic receptors of the bristle worm *Platynereis dumerilii* [69]. We suggest that a similar process might have resulted in the evolution of the sensory pits of Stenostomidae: while the cells of the fundus evolved from the rhabdomeric photoreceptors, they underwent a shift in function in concert with the loss of opsins and associated pigmented cells.

### Sensory pits are not homologous to the cerebral organs of Nemertea

The similarity of the sensory pits of Stenostomidae and the cerebral organs of Nemertea has been used in the past as morphological evidence of their close relationship [13, 35]. Although the level of the integration of cerebral organs and brains varies from one nemertean clade to another [36–39], the organs represent anatomically independent entities that are connected to the brain by specialized nerves. The cerebral organs themselves are composed of the ciliated canal, lined with epidermal multi-ciliated cells, and the inner part, which includes neurons and neuroglandular cells [35, 41, 42]. From those cell types, only the epidermal ciliated cells are in contact with the outer environment, while none of the cells in nemertean cerebral organs show extensive microvilli. In *Stenostomum*, the fundus of the pit, which remains in contact with the outside milieu, is composed of parts of the highly modified neurons, there are no (or only very few) ciliated cells and no neuroglandular cells within the organ. In fact, there is not a single cell type that could be homologized between cerebral organs and sensory pits based on their shared ultrastructure, position, or connectivity. Therefore, the detailed analysis of the cell type diversity and organization in the sensory pits of *Stenostomum* and cerebral organs of nemerteans indicate that both organs show only superficial similarity.

Our investigation of gene expression in the sensory pits of *Stenostomum* also did not reveal any strong similarities in the transcriptional control of the development of both organs. From the tested six transcription factors that are expressed in the cerebral organs of nemertean *Lineus ruber* [42], only one, *pax4/6*, showed expression in the developing sensory pits. An association of *pax4/6* expression with the sensory pits of *Stenostomum* has already been reported before based on the transient expression of a GFP construct under a *pax6* promotor in the sensory pits of *S. leucops* [70] and Pax6 antibody cross-reactivity in *Stenostomum virginianum* [64]. However, as discussed in the previous section, the expression of *pax4/6* in the S*tenostomum* sensory pits might be a result of the evolutionary origin of the fundus-forming cells from rhabdomeric photoreceptors, rather than homology with the nemertean cerebral organs. All in all, both our morphological and molecular evidence argues against a common evolutionary origin of the sensory pits of *Stenostomum* and the cerebral organs of nemerteans.

### Convergent, recurring evolution of sensory pits in flatworms

Sensory pit-like organs are present in several groups of flatworms, e.g., microstomids [13, 14], prorhynchids [15–19], prolecithophorans [20], triclads [21–23], and bothrioplanids [24]. Is it possible that sensory pits were ancestrally present in flatworms and subsequently lost in some lineages? To explore this possibility, we assumed the primary homology of all types of ciliated/sensory pits present in different groups of flatworms and mapped their presence or absence on the flatworm phylogeny (Fig. 8). The maximum-likelihood-based ancestral state reconstruction clearly showed that sensory pits evolved at least six times independently in the platyhelminths. If indeed the sensory pits of stenostomids are derived from photoreceptors as we argued above, the analogy of sensory pits of *Stenostomum* and ciliated pits of other flatworms is not surprising. In the case of at least some microstomids [14, 71], triclads [22, 23], and prolecithophorans [20] the ciliated pits co-occur with the eye spots, indicating that both organs are not directly homologous. Moreover, the pits of rhabditophorans are in fact heavily ciliated and lack extensive microvilli [14, 17], unlike the sensory pits of *Stenostomum*. It is therefore quite likely that organs such as the ciliated pits of rhabditophorans have evolved many times independently, given their apparent simplicity and the fact that external sensory cilia are present in most animals with external ciliation and often provide a receptive surface for diverse stimuli. Accumulation of such sensory ciliated cells within a single sensory pit is plausible from the point of morphological evolvability. Indeed, organs resembling ciliated sensory pits can be found in many distantly related animal clades [72–76], thus indicating the recurring evolution of the ciliated pit-like organs not only in rhabditophorans but across animal phylogeny.

## Conclusions

The sensory pits of the catenulid *Stenostomum brevipharyngium* lack ciliation and instead are mostly composed of sensory neuron-like cells with extensive microvilli and cell nuclei located deeply in the brain lobes. The ultrastructure, spatial arrangement, and position of those cells, together with the fact that they express the bilaterian eye marker *pax4/6*, suggest that they could be derived from ancestral rhabdomeric photoreceptors that have undergone a concomitant functional switch to mechano- or chemoreception. Although in the past, the sensory pits of flatworms have been homologized with the cerebral organs of nemerteans, we neither found morphological nor molecular evidence in support of their homology. Instead, mapping of the head sensory structures on the flatworm phylogeny indicates that organs similar to the sensory pits have evolved many times independently in Platyhelminthes.

## Methods

### Animal culture and amputations

The animals were ordered in 2010 from Connecticut Valley Biological Supply as *Stenostomum* sp. and since then maintained in the laboratory cultures. Species identification followed the key for the American species of *Stenostomum* by JW Nuttycombe and AJ Waters [8]. The worms were kept in Petri dishes with Chalkley’s Medium (CM) in darkness at 20 °C and fed ad libitum with unicellular protist *Chilomonas* sp. Prior to the fixation or amputations, the worms were transferred to fresh CM without food and starved for at least 12 h.

For the amputation experiment the worms were anesthetized with 1% (mass to volume) MgCl_2_ hexahydrate in CM for ca. 10 min. The worms were cut under the dissecting scope with an eyelash at the level of the pharynx and immediately transferred to fresh CM. The regenerates were washed a few times with CM, to remove the anesthetic medium and then kept in darkness at 20 °C.

### Electron microscopy

For the SEM investigation, the worms were starved, anesthetized in 1.44% MgCl_2_ hexahydrate in CM (m:v) for 10 min and fixed for 1 h at room temperature in 4% formaldehyde dissolved in PBS with 0.1% Tween-20 (PTw). Then, the animals were washed a few times in PTw and dehydrated in 100% ethanol. An automatic critical point dryer (Leica EM CPD300) was used to perform critical point drying. After the samples were dehydrated, they were transferred into a container to avoid losing them due to their very small size. These containers were placed into a larger container filled with 100% ethanol and placed in the critical point dryer. 18 cycles of ethanol/liquid carbon dioxide (CO_2_) exchange were performed to quantitatively remove the ethanol. The CO_2_ was then slowly heated to 31 °C with a pressure of 74 bar. It is essential to perform this phase transfer of CO_2_ very slowly in order to avoid capillary forces or volume changes that would damage delicate morphological structures. Dried samples were sputter-coated using the Safematic CCU-010 HV with a 6 nm thick Au layer. The samples were targeted and imaged inside a FIB-SEM (Crossbeam 540, Carl Zeiss Company). The images were acquired with the SEM at 1.5 kV with the secondary electrons (SE2) detector.

For the TEM investigation, the worms were starved, anesthetized in 1.44% MgCl_2_ hexahydrate in CM (m:v) for 10 min, and fixed for 1 h in Shigenaka fixative [77], modified after [78], with pH adjusted to 7.2. Then, the animals were rinsed in ddH_2_0, dehydrated in the acetone series (1 × 50%, 2 × 70%, × 90% 3 × 100%), and left overnight in 1:1 acetone to epoxy resin mixture (EPON; Sigma‐Aldrich). Animals were washed 3 times with 100% epoxy resin (1 h each wash) and embedded in a resin disc. Ultrathin Sects. (80 nm) were cut using a Leica ultracut UCT microtome and contrasted using lead citrate. The specimens were examined with a Zeiss Libra 120 energy filter transmission electron microscope using a Tröndle 2 × 2 k highspeed camera with ImageSP software (Tröndle). Image processing was performed with ImageSP and Image Composite Editor.

### Confocal laser microscopy

The animals for antibody and phalloidin staining were starved, anesthetized in 1.44% MgCl_2_ hexahydrate in CM (m:v) for 10 min, and fixed for 1 h at room temperature in 4% formaldehyde dissolved in PTw. Then the worms were washed a few times in PTw to remove the fixative and kept in PTw in 4 °C for short-term storage. Prior to the antibody staining the worms were washed 3 times in PBT (1 × PBS + 0.1% bovine serum albumin + 0.1% Triton X) and incubated for 30 min at room temperature in the PBT + 5% normal goat serum (PBT + NGS). Then, the worms were incubated overnight at 4 °C in primary antibodies dissolved in PBT + NGS. The following primary antibodies were used: mouse anti-tyrosinated tubulin, Sigma T9028 (dissolved at 1:500); and rabbit anti-serotonin (5HT), Sigma S5545 (dissolved at 1:300). On the following day the worms were washed several times in PBT, incubated for 30 min at room temperature in the PBT + NGS and incubated overnight at 4 °C in secondary antibodies dissolved in PBT + NGS. The following secondary antibodies were used: goat anti-mouse, conjugated with Alexafluor488, Thermo Fisher A-11001; and goat anti-rabbit, conjugated with Alexafluor647, Thermo Fisher A-21244; both at the concentration 1:250. On the following day the animals were washed a few times in PBT and then in PTw and incubated for 40 min at room temperature in nuclear stain dissolved in PTw, either DAPI (at 1:1000) or Hoechst 33,342 (at 1:5000). Afterwards, the worms were washed in PBT and incubated for 1 h at room temperature in Phalloidin, conjugated with Alexafluor555, Thermo Fisher A34055 (10U/ml dissolved in PBT). Finally, the animals were washed in PBS, mounted in FluoromountG, and left overnight at 4 °C to allow tissue clearance and hardening of the mounting medium. The mounted specimens were investigated with the Olympus IX83 microscope with a spinning disc Yokogawa CSUW1-T2S scan head. The obtained confocal Z-stacks were processed for contrast and brightness and analyzed in Fiji [79].

### Generation of the reference transcriptome

Thousands of *Stenostomum brevipharyngium* worms were pooled, including different stages of asexual reproduction and anterior and posterior regeneration, and the total RNA was extracted using the Trizol method. The concentration and integrity of the extracted RNA were measured on BioAnalyzer with Agilent RNA 6000 Nano Kit. After performing poly-A selection, the Illumina cDNA library was generated with the NEBNext Ultra II RNA kit (amplified with 14 PCR cycles), and the library was sequenced on an Illumina NovaSeq 6000 at the Dresden Concept Genome Center to a depth of 300 million paired-end reads with 100 bp length. The raw reads of the reference transcriptome have been uploaded to Sequence Read Archive (BioProject ID PRJNA1004231). Our established and validated de novo transcriptome assembly pipeline [47] was used for quality control and transcriptome assembly (with Trinity Assembler v2.2.0 [80]). The assembled transcriptome has been deposited at the Zenodo data repository and can be publicly accessed online (doi: 10.5281/zenodo.8239273).

### Gene search

Coding sequences for analyzed transcription factors were identified in the transcriptome of *S. brevipharyngium* with the reciprocal TBLASTN search using orthologous protein sequences from *L. ruber* (Additional file 1: Table S1). Sequences of all of the newly identified genes were translated into proteins using Geneious Prime (v2023.0.3) and aligned with reference sequences from other animals (Additional file 1: Table S3) with Muscle Alignment 5.1 implemented in Geneious Prime. The alignments were masked with Geneious Prime to remove sites containing more than 50% gaps and analyzed with FastTree v2.1.11 implemented in Geneious Prime. The protein trees are available as Supplementary Materials (Additional file 2: Figs. S1–S5). All newly obtained sequences were submitted to GenBank (Accession numbers OR036955–OR036965).

The search for opsin sequences followed a similar approach, however, we use multiple query sequences (GenBank accession numbers are provided for each protein): xenopsin from flatworm *Prostheceraeus crozieri* (QEQ50488.1), opsin from flatworm *Dugesia japonica* (CAD13146.1), as well as r-opsin (AGJ70280.1) and c-opsin (ADZ24786.1) from a brachiopod *Terebratalia transversa*. We blasted the query sequences not only against the transcriptome of *S. brevipharyngium* but also against publicly available transcriptomes of *Stenostomum leucops* (NCBI BioProject: PRJNA276469) and *Paracatenula* sp. (NCBI BioProject: PRJEB31702). The obtained BLAST hits were translated into protein sequences in Geneious Prime and BLASTed against NCBI protein database to identify potential opsin paralogs (Additional file 1: Table S1).

### Gene cloning and riboprobe synthesis

The total RNA was extracted from ca. 250 worms at different stages of asexual reproduction and regeneration using the Machery-Nagel NucleoSpin RNA XS kit. The NEB ProtoScript II First Strand cDNA Synthesis Kit was used for cDNA library generation. Fragments of the candidate genes were amplified from the cDNA library in two rounds of PCR (each with 25 cycles, with an annealing temperature of 69 °C) using NEB Q5 High-Fidelity DNA Polymerase and the listed gene-specific primer pairs (Additional file 1: Table S4). The length of the PCR products was analyzed via gel electrophoresis and amplicons of the expected length were cloned into the pPR-T4p-2.0 vector and transformed into competent *Escherichia coli* cells for amplification. Plasmid DNA was isolated and sequenced to confirm the identity of the cloned gene sequences. All plasmids are available upon request to the authors. The digoxigenin-labeled riboprobes were synthesized using T7 polymerase (NEB, M0251L) and precipitated with LiCl. The purified probes were dissolved in a hybridization buffer and stored at − 80 °C.

### *RNA *in situ hybridization

The protocol for colorimetric in situ hybridization followed the one from Hejnol 2008 [81] with minor modifications. The animals were relaxed in 1.44% MgCl_2_ for 10 min and fixed in 4% formaldehyde in PTw for 1 h at room temperature. We omitted the triethanolamine and acetic anhydrate treatments and we performed hybridization over the weekend (ca. 70 h) at 67 °C. The stained worms were mounted in 80% Glycerol in PTw and examined with the Zeiss Axiophot upright microscope.

For fluorescent RNA in situ hybridization chain reaction v3.0 (HCR) [82], we used the Özpolat Lab’s in situ probe generator [83] for the design of DNA probe oligo pools (Additional file 1: Table S5). The probe pools were ordered from Integrated DNA Technologies and hybridized using the published HCR protocol by Piovani et al. [84]. The stained specimens were mounted in FluoromountG (Thermo Fischer, 00–4958-02) and imaged on an Olympus IX83 microscope with a spinning disc Yokogawa CSUW1-T2S scan head. The obtained confocal Z-stacks were processed for contrast and brightness and analyzed in Fiji [79].

### Ancestral state reconstruction

We collected 57 *18S*, 56 *28S*, 12 *ITS-5.8S*, and 32 *COI* sequences from representatives of all major flatworm clades (Additional file 1: Table S2). The corresponding sequences for *S. brevipharyngium* have been retrieved from our reference transcriptome and submitted to GenBank under accession numbers OR432512, OR432521, OR436478, and OR436511. Each gene was aligned separately with MAFFT (version: v7.520 [85], parameters: –maxiterate 1000 –globalpair), and columns with more than 60% missing data were removed using ClipKit (version: 1.4.1 [86], parameters: -m gappy -g 0.6). The alignments were then combined using AMAS (commit: 2e93d31) [87] resulting in a final multiple sequence alignment 5610 bp in length with 36.6% missing data and 3245 parsimony informative sites. ModelFinder [88] was used to determine the best-fitting substitution model for each partition (*18S* and *28S*: GTR + F + I + G4, *ITS-5.8S*:TVM + F + G4, and *COI*: K3Pu + F + G4) and the phylogeny was inferred with these partitions using IQ-TREE (version: 2.2.2.7) [89]. One thousand Ultrafast Bootstraps [90] were calculated to assess node support.

To transform the resulting phylogeny to be ultrametric we scaled it with the penalized likelihood approach [91] implemented in the Chronos function of the R package ape (Version: 5.6–2) [92]. We determined the appropriate smoothing parameter by performing the analysis with values spanning six orders of magnitude and comparing the likelihood. Since we found the likelihood plateaued towards low values, we chose 0.001 as the optimum, but note that the inference was robust to the choice of smoothing parameter value. Next, we fit two MK-models to the data, one with equal rates (ER) for gains and losses of the sensory pits and one with all rates different (ARD). We performed a likelihood ratio test to determine which model was a better fit to the data, finding that the simpler ER model was preferred (df:1, *p*-value = 0.97). Then we used stochastic character mapping [93] implemented in the R package phytools (Version: 1.0–1) [94] to simulate 10,000 character histories of the sensory pits using the transition matrix from the ER model. We used the Maximum-likelihood implementation of the method, which samples histories from the most likely transition matrix. We performed 10,000 iterations of burn-in, followed by 100,000 iterations where we retained every 10th character history. Finally, we summarized the ancestral state as the proportion of stochastic histories with either pits present or absent.

### Supplementary Information

**Additional file 1: Table S1. **Results of the reciprocal BLAST search in the transcriptomes of catenulids. **Table S2. **Accession numbers of the nucleotide sequences used to reconstruct the phylogeny of flatworms. **Tables S3. **Accession numbers of the protein sequences used to identify studied genes with the phylogentic approach. **Table S4. **Primers pairs used to clone genes of interest from cDNA library. **Table S5. **Pools of HCR probes.

**Additional file 2: Fig. S1. **Phylogenetic analysis of PRD-class homeobox transcription factors.** Fig. S2. **Phylogenetic analysis of nuclear receptor subfamily 2. **Fig. S3. **Phylogenetic analysis of Pax sequences. **Fig. S4. **Phylogenetic analysis of Dach sequences. **Fig. S5. **Phylogenetic analysis of Emx sequences. **Fig. S6. **Molecular phylogeny of flatworms inferred with a maximum likelihood approach from the concatenated *18S*, *28S*, *ITS-5.8S*, and *COI* datasets.

## Data Availability

The raw reads of the reference transcriptome of *S. brevipharyngium* are deposited at NCBI Sequence Reads Archive (BioProject ID PRJNA1004231). The assembled transcriptome is available at Zenodo.org data repository (doi: 10.5281/zenodo.8239273). Newly obtained nucleotide sequences of analyzed transcription factors and molecular phylogenetic markers are deposited in GenBank (accession numbers OR036955–OR036965, OR432512, OR432521, OR436478 and OR436511). All other data generated or analyzed during this study are included in this published article and its supplementary materials.
