# Supplementary material for: Convergent evolution of the sensory pits in and within flatworms
Source: BMC Biol. 2023 Nov 22;21:266. doi: 10.1186/s12915-023-01768-y (PMC10664644; doi:10.1186/s12915-023-01768-y)
Supplement: Supplementary file 2 — Additional file 2: Fig. S1. Phylogenetic analysis of PRD-class homeobox transcription factors. Fig. S2. Phylogenetic analysis of nuclear receptor subfamily 2. Fig. S3. Phylogenetic analysis of Pax sequences. Fig. S4. Phylogenetic analysis of Dach sequences. Fig. S5. Phylogenetic analysis of Emx sequences. Fig. S6. Molecular phylogeny of flatworms inferred with a maximum likelihood approach from the concatenated 18S, 28S, ITS-5.8S, and COI datasets. [file 12915_2023_1768_MOESM2_ESM.docx]

**Additional file 2**

**Convergent evolution of the sensory pits in and within flatworms**

Ludwik Gąsiorowski^1*^, Isabel Lucia Dittmann^2^, Jeremias N. Brand^1^, Torben Ruhwedel^3^, Wiebke Möbius^3^, Bernhard Egger^2^, Jochen C. Rink^1*^

^1^ Department of Tissue Dynamics and Regeneration, Max Planck Institute for Multidisciplinary Sciences, Am Fassberg 11 37077 Göttingen, Germany.

^2^ Institut für Zoologie, Universität Innsbruck, Technikerstraße 25 6020 Innsbruck, Austria.

^3^ Electron Microscopy Core Unit, Max Planck Institute for Multidisciplinary Sciences, Hermann-Rein-Str. 3, 37075 Göttingen, Germany


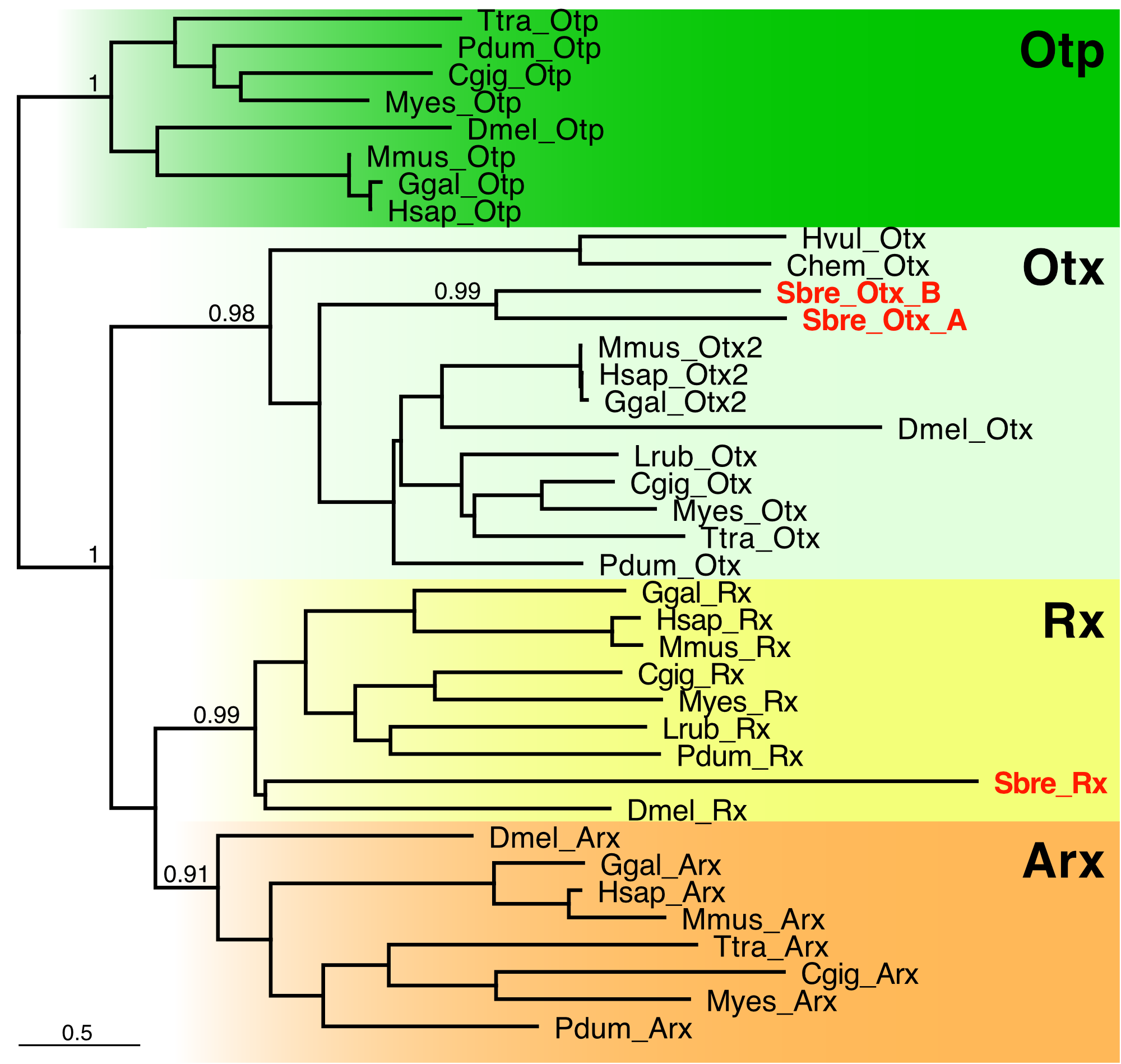


**Fig. S1.** Phylogenetic analysis of PRD-class homeobox transcription factors. SH-like support values are shown for the important nodes. The scale bar on the lower right corner shows the amino acid substitution rate per site. Sequences from *S. brevipharyngium* are marked in red. For abbreviation and source of other sequences see table S3.


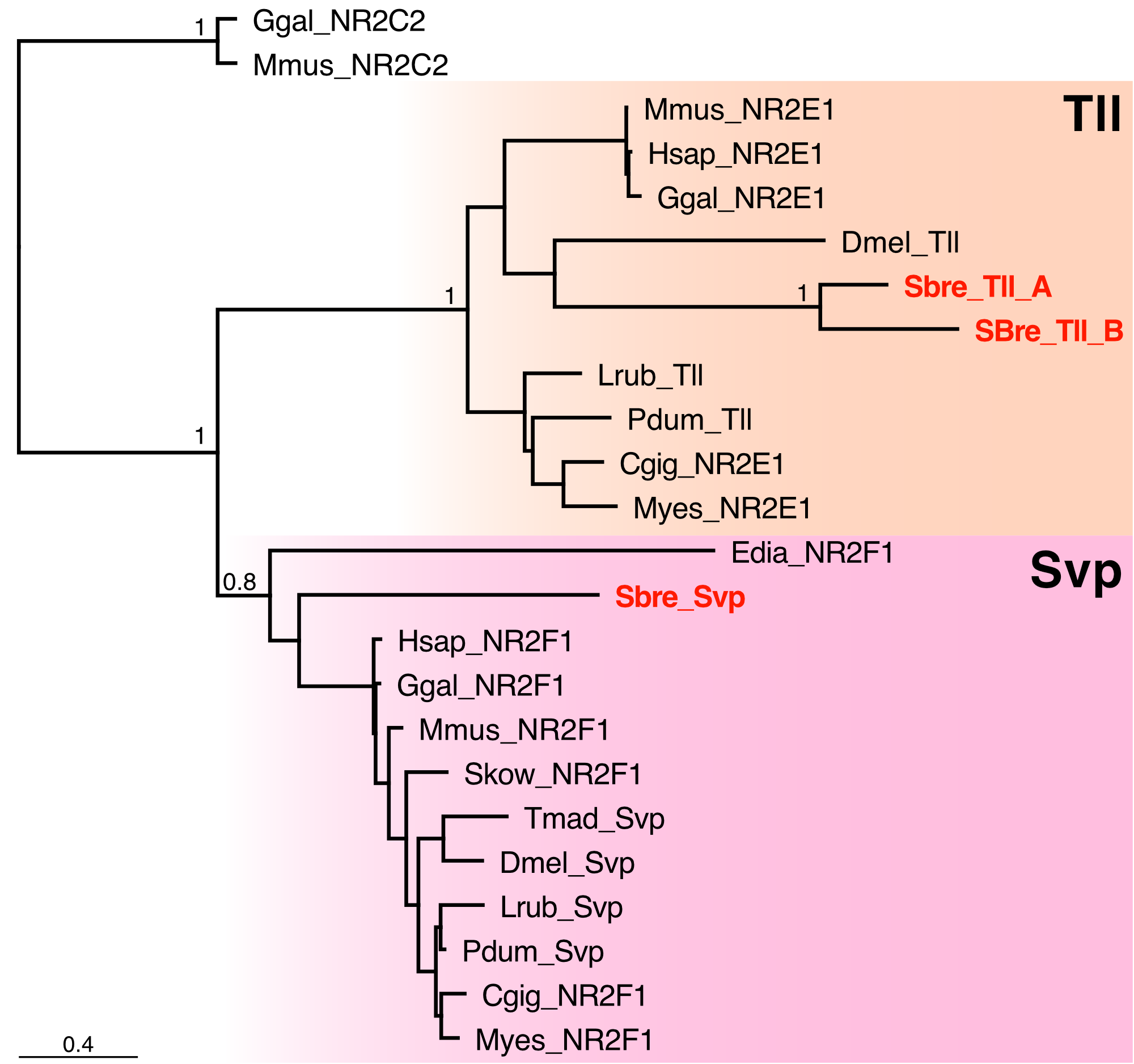


**Fig. S2.** Phylogenetic analysis of nuclear receptor subfamily 2. SH-like support values are shown for the important nodes. The scale bar on the lower right corner shows the amino acid substitution rate per site. Sequences from *S. brevipharyngium* are marked in red. For abbreviation and source of other sequences see table S3.


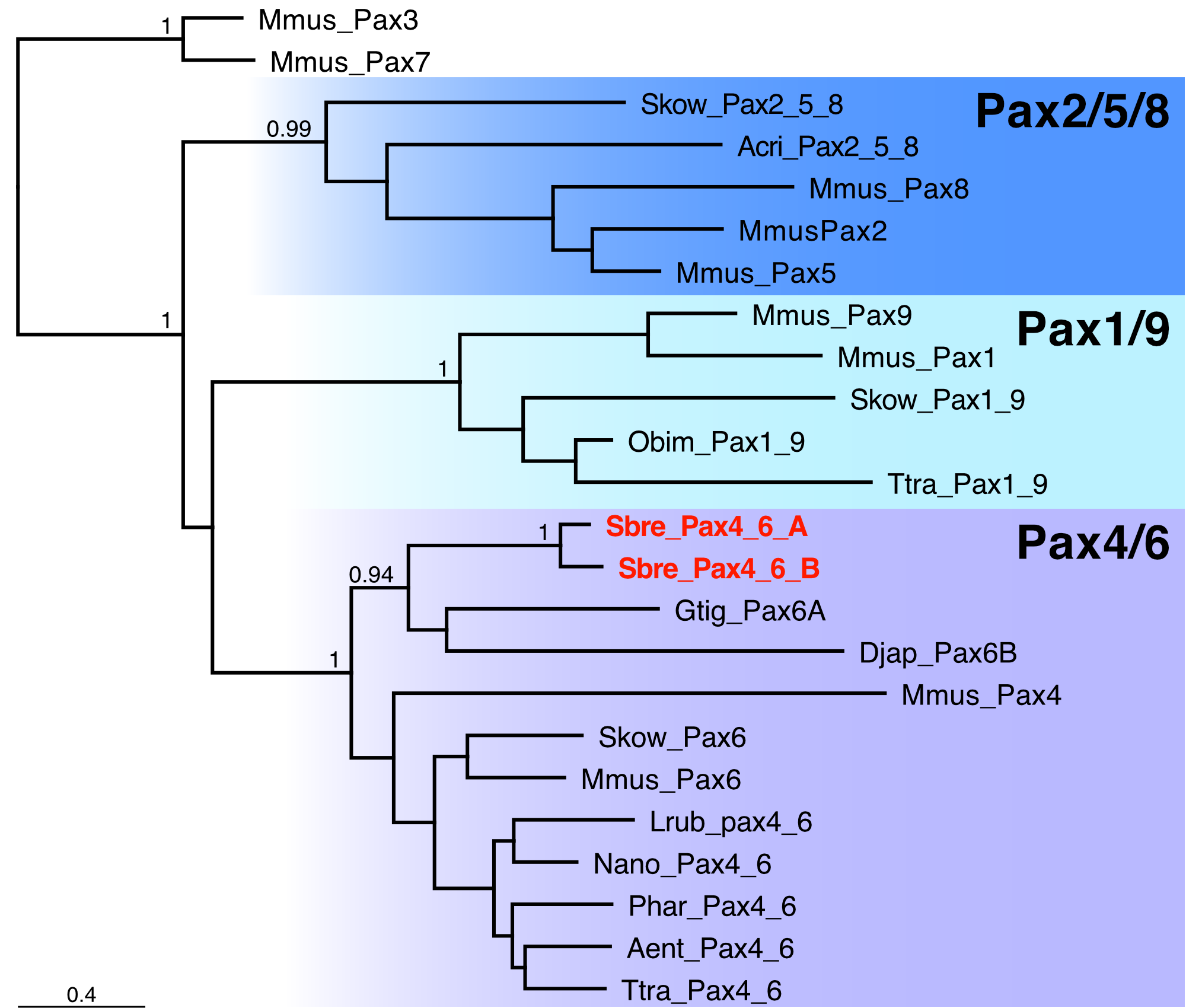


**Fig. S3.** Phylogenetic analysis of Pax sequences. SH-like support values are shown for the important nodes. The scale bar on the lower right corner shows the amino acid substitution rate per site. Sequences from *S. brevipharyngium* are marked in red. For abbreviation and source of other sequences see table S3.


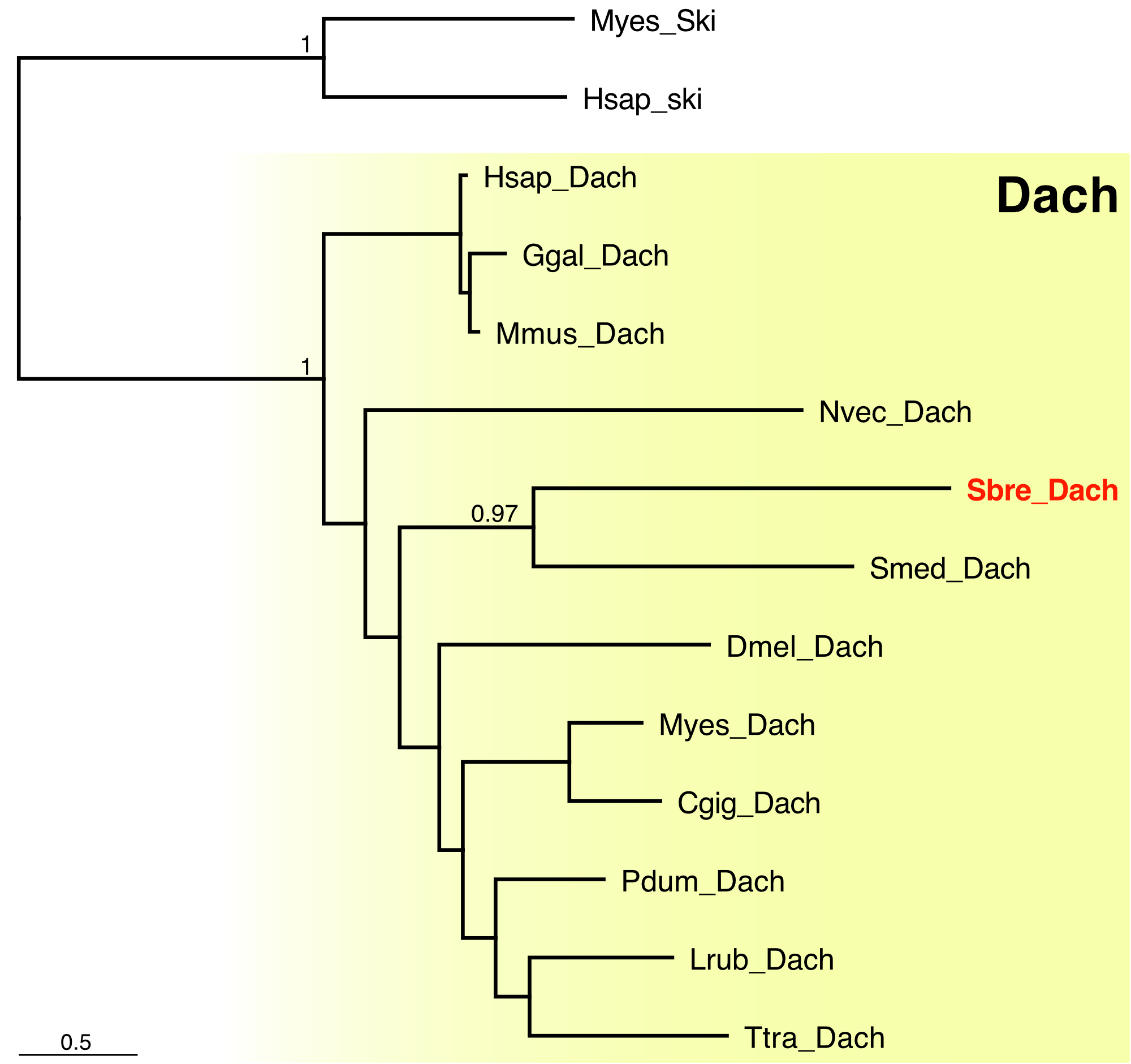


**Fig. S4.** Phylogenetic analysis of Dach sequences. SH-like support values are shown for the important nodes. Scale bar on the lower right corner shows the amino acid substitution rate per site. The sequence from *S. brevipharyngium* is marked in red. For abbreviation and source of other sequences see table S3.


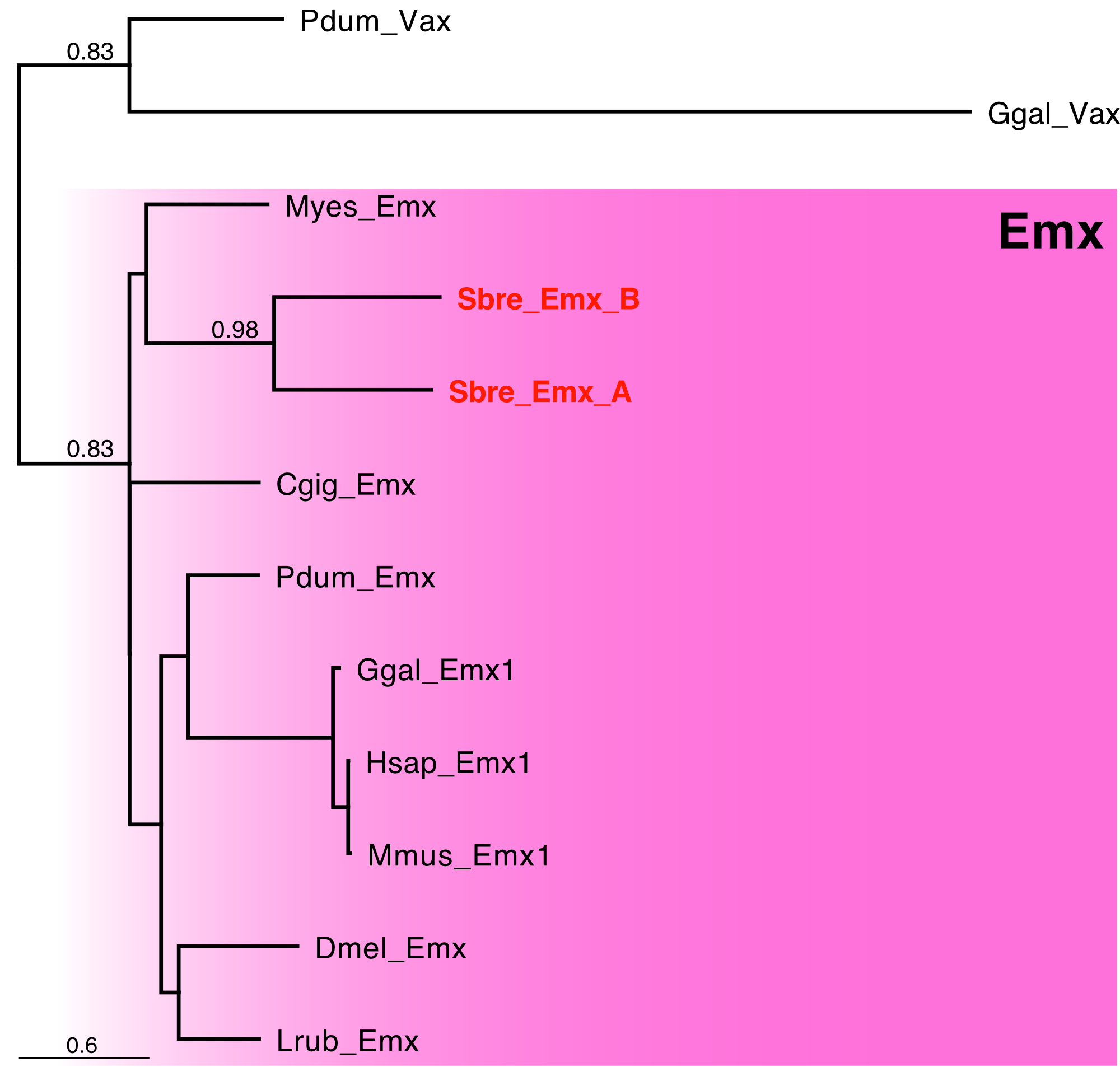


**Fig. S5.** Phylogenetic analysis of Emx sequences. SH-like support values are shown for the important nodes. The scale bar on the lower right corner shows the amino acid substitution rate per site. The sequence from *S. brevipharyngium* is marked in red. For abbreviation and source of other sequences see table S3.


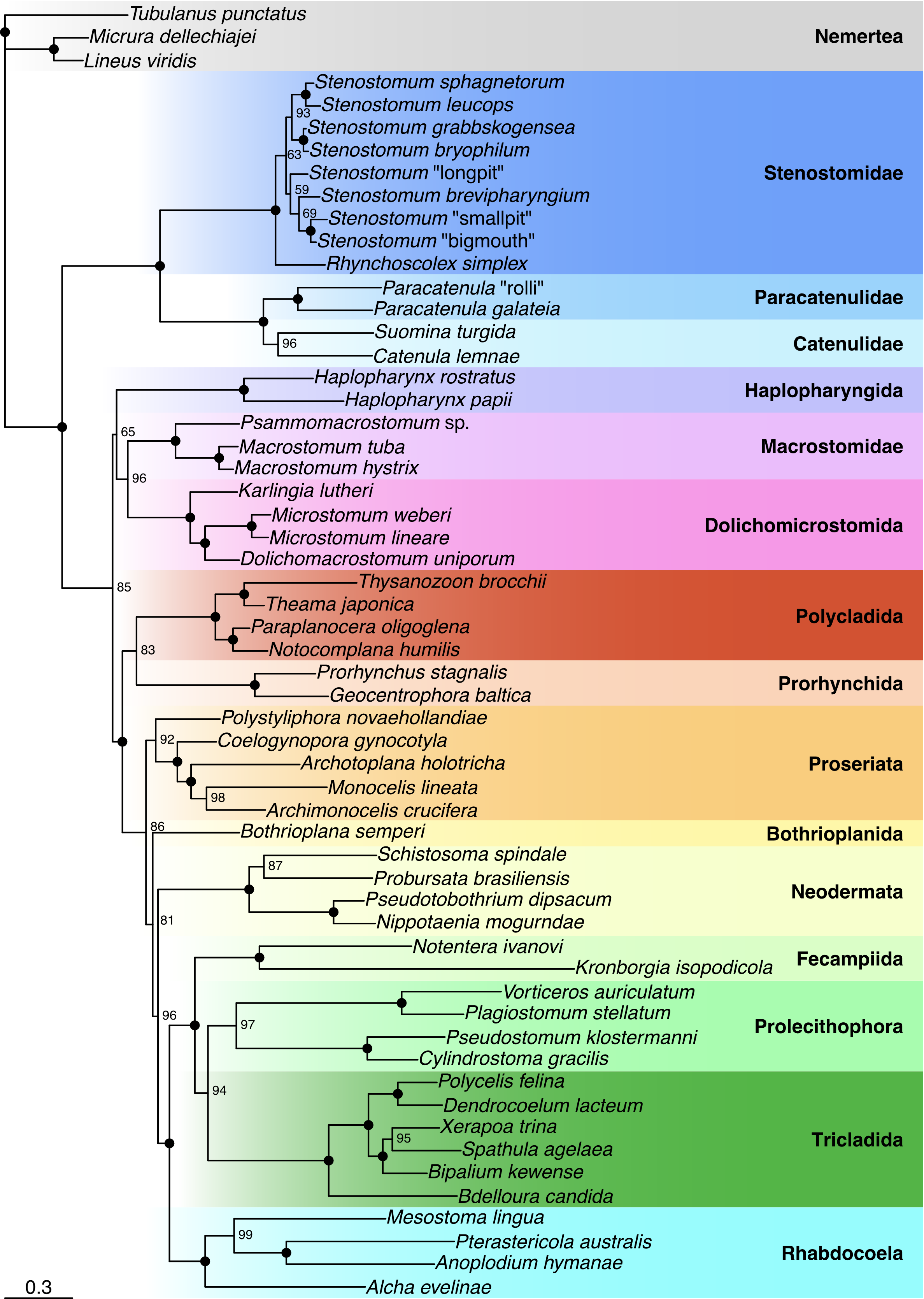


**Fig. S6.** Molecular phylogeny of flatworms inferred with a maximum likelihood approach from the concatenated *18S*, *28S*, *ITS-5.8S,* and *COI* datasets. Values above branches indicate ultrafast bootstrap support (filled dots when 100).
